# Targeting lysine demethylase 6B ameliorates ASXL1 truncation–mediated myeloid malignancies in preclinical models

**DOI:** 10.1172/JCI163964

**Published:** 2024-01-02

**Authors:** Guo Ge, Peng Zhang, Pinpin Sui, Shi Chen, Hui Yang, Ying Guo, Ivan P. Rubalcava, Asra Noor, Caroline R. Delma, Joel Agosto-Peña, Hui Geng, Edward A. Medina, Ying Liang, Stephen D. Nimer, Ruben Mesa, Omar Abdel-Wahab, Mingjiang Xu, Feng-Chun Yang

**Affiliations:** 1 Department of Cell Systems and Anatomy,; 2 Mays Cancer Center,; 3 Department of Molecular Medicine, and; 4 Department of Pathology and Laboratory Medicine, University of Texas Health Science Center at San Antonio, San Antonio, Texas, USA.; 5 Lindsley F. Kimball Research Institute, New York Blood Center, New York, New York, USA.; 6 Sylvester Comprehensive Cancer Center, University of Miami Miller School of Medicine, Miami, Florida, USA.; 7 Human Oncology and Pathogenesis Program, Memorial Sloan Kettering Cancer Center, New York, New York, USA.

**Keywords:** Hematology, Hematopoietic stem cells, Leukemias, Mouse models

## Abstract

*ASXL1* mutation frequently occurs in all forms of myeloid malignancies and is associated with aggressive disease and poor prognosis. ASXL1 recruits Polycomb repressive complex 2 (PRC2) to specific gene loci to repress transcription through trimethylation of histone H3 on lysine 27 (H3K27me3). *ASXL1* alterations reduce H3K27me3 levels, which results in leukemogenic gene expression and the development of myeloid malignancies. Standard therapies for myeloid malignancies have limited efficacy when mutated *ASXL1* is present. We discovered upregulation of lysine demethylase 6B (KDM6B), a demethylase for H3K27me3, in *ASXL1-*mutant leukemic cells, which further reduces H3K27me3 levels and facilitates myeloid transformation. Here, we demonstrated that heterozygous deletion of *Kdm6b* restored H3K27me3 levels and normalized dysregulated gene expression in *Asxl1^Y588X^*Tg hematopoietic stem/progenitor cells (HSPCs). Furthermore, heterozygous deletion of *Kdm6b* decreased the HSPC pool, restored their self-renewal capacity, prevented biased myeloid differentiation, and abrogated progression to myeloid malignancies in *Asxl1^Y588X^*Tg mice. Importantly, administration of GSK-J4, a KDM6B inhibitor, not only restored H3K27me3 levels but also reduced the disease burden in NSG mice xenografted with human *ASXL1*-mutant leukemic cells in vivo. This preclinical finding provides compelling evidence that targeting KDM6B may be a therapeutic strategy for myeloid malignancies with *ASXL1* mutations.

## Abstract 2

Genetic deletion of Kdm6b or pharmacologic inhibition of KDM6B corrects abnormal hematopoietic stem/progenitor cell behavior and prevents Asxl1-driven myeloid malignancy in preclinical models.

## Graphical abstract

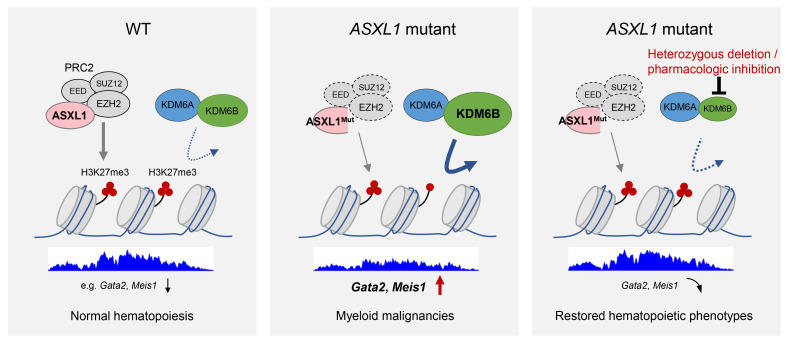

## Introduction

Additional sex combs–like 1 (*ASXL1*) is one of the most frequently mutated genes across the spectrum of myeloid malignancies, including chronic myelomonocytic leukemia (1, 2), myelodysplastic syndrome (MDS) (3, 4), myeloproliferative neoplasms (MPNs) (5, 6), juvenile myelomonocytic leukemia (7, 8), and acute myeloid leukemia (AML) (9, 10). The majority of *ASXL1* alterations are nonsense or frameshift mutations, generating C-terminally truncated proteins (11, 12). Notably, *ASXL1* mutations are associated with aggressive disease and poor prognosis (2, 11). *ASXL1* mutations have also been implicated in clonal hematopoiesis of indeterminate potential (13–15), indicating that *ASXL1* is one of the early genetic events that occur during myeloid transformation. We and others have reported that *Asxl1* loss or transgenic expression of a truncated ASXL1 protein in mice (e.g., *Asxl1^Y588X^*Tg) impairs hematopoietic stem/progenitor cell (HSPC) function and leads to the development of diverse myeloid malignancies (16–21), demonstrating a crucial role of ASXL1 in normal hematopoiesis and its mutation in the pathogenesis of myeloid malignancies. Therapies targeting *ASXL1* mutation–mediated epigenetic alterations may improve the survival of patients with *ASXL1* mutation–associated myeloid malignancies.

ASXL1 regulates gene expression epigenetically by cooperating with other chromatin modifiers, such as Polycomb repressive complex 2 (PRC2) (22), BAP1 (23), and cohesin complex proteins (24). ASXL1 interacts directly with the core subunits of PRC2 (EZH2, SUZ12, and EED), and recruits the PRC2 complex proteins to specific gene loci to repress transcription through trimethylation of histone H3 on lysine 27 (H3K27me3) (22). Loss of *Asxl1* in hematopoietic cells leads to the dysregulated expression of genes critical for HSPC functions, which is correlated with the global reduction of H3K27me3 (16, 17, 25). Inoue and colleagues reported that stable expression of truncating ASXL1 mutants impairs PRC2 function and results in the global reduction of H3K27me3 (18). These studies highlight the significance of H3K27me3 regulation in normal hematopoiesis and *ASXL1* mutation–mediated myeloid transformation. However, the precise nature of the mutant *ASXL1*–induced epigenetic dysregulation that contributes to leukemogenesis remains unclear.

Lysine demethylase 6B (KDM6B, also known as JMJD3) is a Jumonji C domain–containing histone demethylase that specifically removes di- and trimethyl groups from H3K27 (H3K27me2/me3) (26, 27). KDM6B is involved in various biological processes, including inflammatory responses (28), development (29, 30), stress-induced senescence (31), and differentiation (32). Mallaney and colleagues demonstrated that *Kdm6b* is necessary for the self-renewal of hematopoietic stem cells (HSCs) in response to inflammatory and proliferative stress, and loss of *Kdm6b* leads to the depletion of phenotypic and functional HSCs in adult mice (33). However, multiple recent studies have shown an oncogenic effect of KDM6B overexpression in hematologic malignancies, including MDS (34), AML (35), multiple myeloma (36), and lymphoid malignancies (37–39). Thus, the balance of the KDM6B levels is important for normal hematopoiesis and tumor suppression.

Here, we show that KDM6B is markedly upregulated in *Asxl1^Y588X^*Tg HSPCs, while the protein levels of EZH2, SUZ12, EED, and KDM6A (UTX, H3K27me3 demethylase) remain unchanged. Heterozygous deletion of *Kdm6b* in *Asxl1^Y588X^*Tg mice restores the HSC pool, normalizes the biased myeloid differentiation, and prevents the development of ASXL1^aa1–587^-driven myeloid malignancies. Transcriptional analysis showed that heterozygous deletion of *Kdm6b* in *Asxl1^Y588X^*Tg normalizes the majority of upregulated genes critical for HSC function and leukemia development, correlating with restored H3K27me3 levels. Notably, the application of GSK-J4 inhibits the mutant ASXL1–mediated leukemic cell growth and prevents the development of mutant ASXL1–driven myeloid malignancies. This study provides solid preclinical evidence that KDM6B may serve as a therapeutic target for patients with *ASXL1* mutation–associated myeloid malignancies.

## Results

### KDM6B is upregulated in Asxl1-mutant cells.

**Figure 1:**
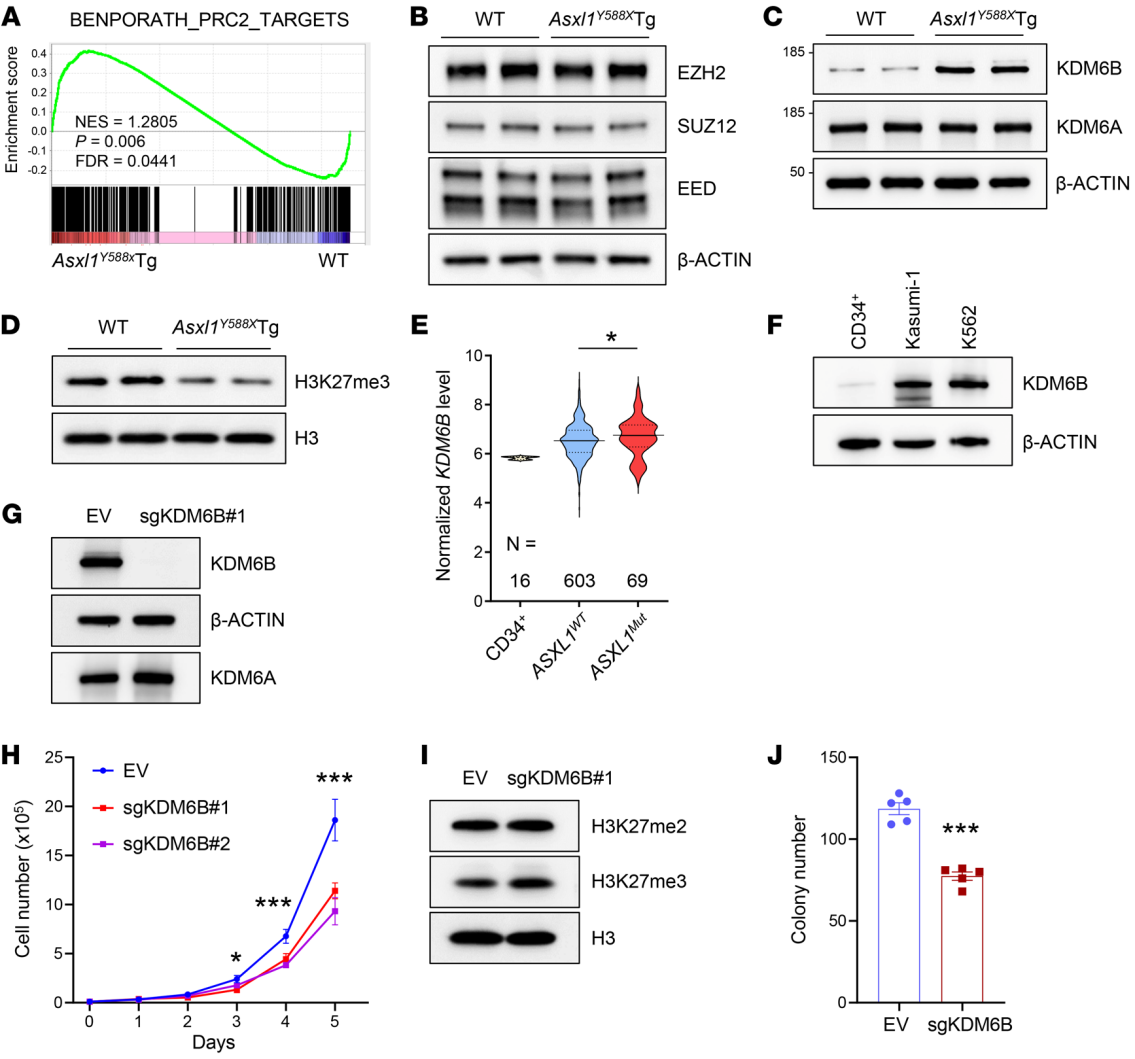
KDM6B is upregulated in *Asxl1*-mutant cells. (**A**) Gene set enrichment analysis (GSEA) shows that PRC2 target genes are upregulated in *Asxl1^Y588X^*Tg HSPCs. The normalized enrichment score (NES), *P* value, and FDR are shown. (**B**) Western blot analysis of indicated members of the PRC2 complex in BM cells from WT and *Asxl1^Y588X^*Tg mice. (**C**) The protein levels of histone H3K27 demethylases KDM6A and KDM6B were measured in WT and *Asxl1^Y588X^*Tg BM cells. (**D**) Western blot showing the level of H3K27me3 in *Asxl1^Y588X^*Tg BM cells. H3 was used as a loading control. (**E**) Normalized *KDM6B* RNA level in CD34^+^ cells and AML patients with or without *ASXL1* mutations from the Beat AML 2.0 database. (**F**) Western blot analysis of KDM6B in *ASXL1*-mutant leukemia cell lines and human BM CD34^+^ cells. Kasumi-1, *ASXL1* G646WfsX12; and K562, *ASXL1* Y591X. (**G** and **I**) Western blot analysis of KDM6B (**G**) and H3K27me2/3 (**I**) levels in K562 cells after expressing empty vector (EV) or sgRNA targeting KDM6B (sgKDM6B). (**H**) Proliferation curves of K562 cells transfected with EV and sgKDM6B. (**J**) Colony-forming unit assay using K562 cells was assessed in semisolid medium. Data were derived from 3 independent experiments and represent the mean ± SEM. *P < 0.05 and ***P < 0.001, by Mann-Whitney *U* test (**E**), 1-way ANOVA with Tukey’s multiple-comparison test (**H**), or unpaired Student’s *t* test (**J**).

Inoue et al. reported that truncating *ASXL1* mutation upregulated the expression of *Hoxa9* and microRNA-125a through decreased H3K27me3 (18). To assess whether the dysregulated genes in *Asxl1^Y588X^*Tg HSPCs are associated with H3K27me3, we revisited our RNA-Seq data (PRJNA388673) (19) and observed an upregulation of the PRC2 target genes in *Asxl1^Y588X^*Tg HSPCs by gene set enrichment analysis (GSEA) (Figure 1A and Supplemental Figure 1, A and B; supplemental material available online with this article; https://doi.org/10.1172/JCI163964DS1), reinforcing the role of H3K27me3 in gene suppression in *Asxl1^Y588X^*Tg HSPCs. We then performed Western blot analysis to determine whether ASXL1 truncation affects the protein levels of PRC2 subunits, including EZH2, SUZ12, and EED, in *Asxl1^Y588X^*Tg bone marrow (BM) cells. The result showed that the levels of EZH2, SUZ12, and EED were comparable between *Asxl1^Y588X^*Tg and WT cells (Figure 1B). Since H3K27me3 levels are maintained by the balance between the activities of histone methyltransferases (EZH2) and demethylases (KDM6A and KDM6B) (40, 41), we next evaluated the protein levels of KDM6A and KDM6B. Notably, the level of KDM6B was dramatically increased in *Asxl1^Y588X^*Tg BM cells compared with WT controls, while the level of KDM6A was comparable between the 2 genotypes of cells (Figure 1C). Further analysis by Western blot confirmed a decreased level of global H3K27me3 in *Asxl1^Y588X^*Tg cells compared with WT controls (Figure 1D). These findings raise the possibility that increased KDM6B is a mediator of decreased H3K27me3 levels and dysregulated gene expression in *Asxl1^Y588X^*Tg HSPCs.

It has been shown that KDM6B is upregulated in MDS and AML (34, 35). By analyzing the Beat AML 2.0 database (42), we found a higher *KDM6B* expression in *ASXL1*-mutated AML patients compared with AML patients with WT *ASXL1* (Figure 1E). Interestingly, the higher level of *KDM6B* correlated with a shorter overall survival in AML patients with *ASXL1* truncation mutations (Supplemental Figure 1C). We next performed Western blots to assess the protein levels of KDM6B in two leukemia cell lines, Kasumi-1 and K562 cells with *ASXL1* truncating mutations as *ASXL1* G646WfsX12 and *ASXL1* Y591X, respectively. KDM6B was highly expressed in Kasumi-1 and K562 cells compared with BM CD34^+^ cells (Figure 1F). To gain insight into the impact of KDM6B overexpression in truncated ASXL1–associated leukemia development, we generated two KDM6B knockout clones in K562 cells using the CRISPR/Cas9 system. Depletion of *KDM6B* by sgKDM6B was confirmed by Western blot analysis (Figure 1G and Supplemental Figure 1D) and sequencing of the *KDM6B* locus in two K562 clones (Supplemental Figure 1E). KDM6A levels were not affected by sgKDM6B (Figure 1G). KDM6B depletion significantly decreased the proliferation of cells (Figure 1H) and increased the global level of H3K27me3 in K562 cells (Figure 1I and Supplemental Figure 1, F and G). Furthermore, a semisolid colony-forming unit cell (CFU-C) assay revealed that KDM6B depletion reduced the frequency of CFU-Cs (Figure 1J). These data indicate that KDM6B is overexpressed in leukemic cells with *ASXL1* mutation, and can be targeted to restore the levels of H3K27me3 and improve HSPC function.

### Heterozygous deletion of Kdm6b blocks the development of ASXL1^aa1–587^–mediated myeloid malignancies.

**Figure 2:**
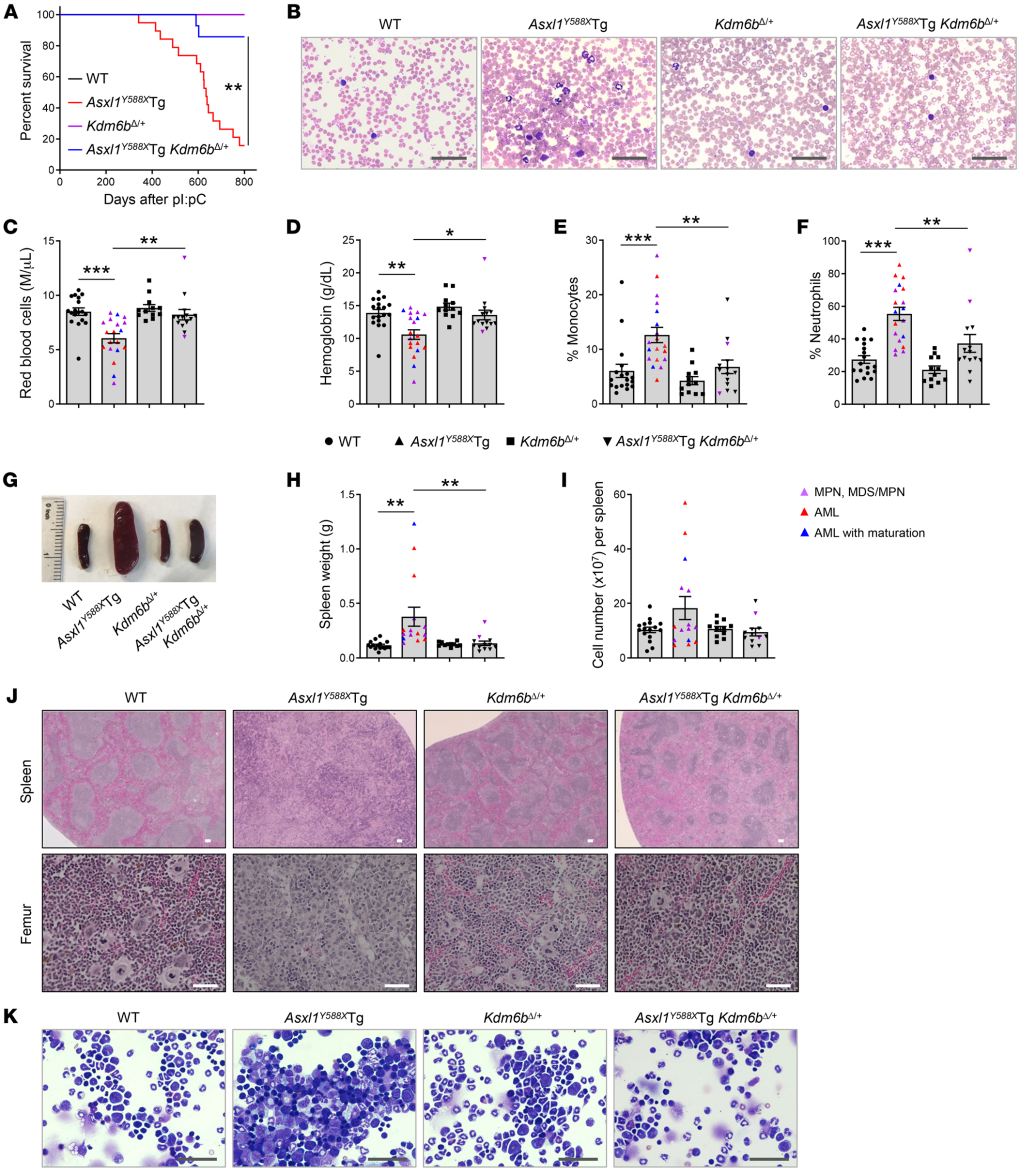
Heterozygous deletion of *Kdm6b* blocks the development of ASXL1^aa1–587^–mediated myeloid malignancies. (**A**) Kaplan-Meier survival curve representing percent survival of *Asxl1^Y588X^*Tg (*n* = 19), *Kdm6b^Δ/+^* (*n* = 12), *Asxl1^Y588X^*Tg *Kdm6b^Δ/+^* (*n* = 14), and WT (*n* = 18) mice. (**B**) May-Giemsa–stained PB smears from representative mice of each genotype. Scale bars: 50 μm. (**C**–**F**) PB counts of red blood cells (**C**), hemoglobin (**D**), and percent of monocytes (**E**) and neutrophils (**F**) in WT (*n* = 18), *Asxl1^Y588X^*Tg (*n* = 19), *Kdm6b^Δ/+^* (*n* = 12), and *Asxl1^Y588X^*Tg *Kdm6b^Δ/+^* (*n* = 14) mice. (**G**) Gross appearance of spleen from representative mice of each genotype. (**H** and **I**) Weight (**H**) and cellularity (**I**) of the spleen from WT (*n* = 16), *Asxl1^Y588X^*Tg (*n* = 15), *Kdm6b^Δ/+^* (*n* = 11), and *Asxl1^Y588X^*Tg *Kdm6b^Δ/+^* (*n* = 12) mice. (**J**) Representative H&E staining of spleen and femur sections. Scale bars: 100 μm (top); 50 μm (bottom). (**K**) May-Giemsa–stained BM cytospins prepared from representative mice of each genotype. Scale bars: 50 μm. Data represent the mean ± SEM. *P < 0.05, **P < 0.01, and ***P < 0.001, by log-rank (Mantel-Cox) test (**A**) or 1-way ANOVA with Tukey’s multiple-comparison test (**C**–**F**, **H**, and **I**).

To evaluate whether deletion of KDM6B could mitigate the development of truncated ASXL1–driven myeloid malignancies, we crossed *Asxl1^Y588X^*Tg with *Mx1Cre^+^* *Kdm6b^fl/+^* mice (43) and generated *Asxl1^Y588X^*Tg *Mx1Cre^+^* *Kdm6b^fl/+^* mice (Supplemental Figure 2A). Deletion of *Kdm6b* (*Kdm6b^Δ/+^*) was induced by polyinosine-polycytidine (pI:pC) and confirmed by PCR, quantitative PCR (qPCR), and Western blot analyses (Supplemental Figure 2, A–D). The survival rate of *Asxl1^Y588X^*Tg *Kdm6b^Δ/+^* was significantly higher than that of *Asxl1^Y588X^*Tg mice (Figure 2A). Consistent with our previous work (19), necropsy of moribund or diseased *Asxl1^Y588X^*Tg mice demonstrated that *Asxl1^Y588X^* mice (19/19, 100%) developed myeloid malignancies after 10 months of age, including MPNs, MDS/MPNs, and myeloid leukemia (Supplemental Table 1). In contrast, only 2 of the *Asxl1^Y588X^*Tg *Kdm6b^Δ/+^* mice (2/14, 14.3%) developed MDS/MPNs, and the remaining *Asxl1^Y588X^*Tg *Kdm6b^Δ/+^* mice exhibited no detectable abnormalities in hematopoiesis (Supplemental Table 2).

Peripheral blood (PB) smears of *Asxl1^Y588X^*Tg mice showed neutrophilia (Figure 2B) with dysplasia present in nucleated red blood cells (e.g., irregular nuclear contours/blebbing and Howell-Jolly bodies) and neutrophils (e.g., abnormal segmentation) (Supplemental Figure 2E), while *Kdm6b^Δ/+^* and *Asxl1^Y588X^*Tg *Kdm6b^Δ/+^* PB smears were morphologically unremarkable as compared with WT controls (Figure 2B). Examination of PB parameters revealed that *Asxl1^Y588X^*Tg mice had decreased red blood cell counts and lower hemoglobin levels compared with WT, while *Asxl1^Y588X^*Tg *Kdm6b^Δ/+^* mice had levels comparable to those of WT mice (Figure 2, C and D). The percentages of monocytes and neutrophils in *Asxl1^Y588X^*Tg mice were significantly higher than those in WT mice, while their percentages in *Asxl1^Y588X^*Tg *Kdm6b^Δ/+^* mice were significantly lower than those in *Asxl1^Y588X^*Tg mice (Figure 2, E and F). In contrast, the percentage of lymphocytes in *Asxl1^Y588X^*Tg mice was significantly decreased compared with that in WT mice, whereas the percentage of lymphocytes in *Asxl1^Y588X^*Tg *Kdm6b^Δ/+^* mice was comparable to that in WT mice (Supplemental Figure 2F). We did not observe a difference of overall number of white blood cells among the 4 genotypes of mice (Supplemental Figure 2G).

Despite the comparable body weights among the 4 groups of mice (Supplemental Figure 2H), *Asxl1^Y588X^*Tg mice exhibited splenomegaly (Figure 2, G and H), consistent with our previous report (19). Deletion of 1 allele of *Kdm6b* normalized spleen sizes and weights of *Asxl1^Y588X^*Tg mice to those of WT mice (Figure 2, G–I). Histologic analysis of H&E-stained BM, spleen, and liver sections revealed that, while *Asxl1^Y588X^*Tg mice demonstrated architectural disruption due to a pronounced myeloid cell infiltration, *Asxl1^Y588X^*Tg *Kdm6b^Δ/+^* mice displayed no obvious histologic abnormalities in these organs (Figure 2J and Supplemental Figure 2, I–K). Analysis of BM cytospin preparations revealed that, in contrast to the marked increase in blasts in *Asxl1^Y588X^*Tg BM, the number of blasts in the BM of *Asxl1^Y588X^*Tg *Kdm6b^Δ/+^* mice was comparable to that of WT mice (Figure 2K). Together, these data suggest that heterozygous deletion of *Kdm6b* in *Asxl1^Y588X^*Tg mice is sufficient to reverse the hematologic abnormalities due to ASXL1^aa1–587^ expression and prevent the progression of ASXL1^aa1–587^-mediated myeloid malignancies.

### Heterozygous deletion of Kdm6b restores ASXL1^aa1–587^–mediated HSC phenotypes and myeloid differentiation.

**Figure 3:**
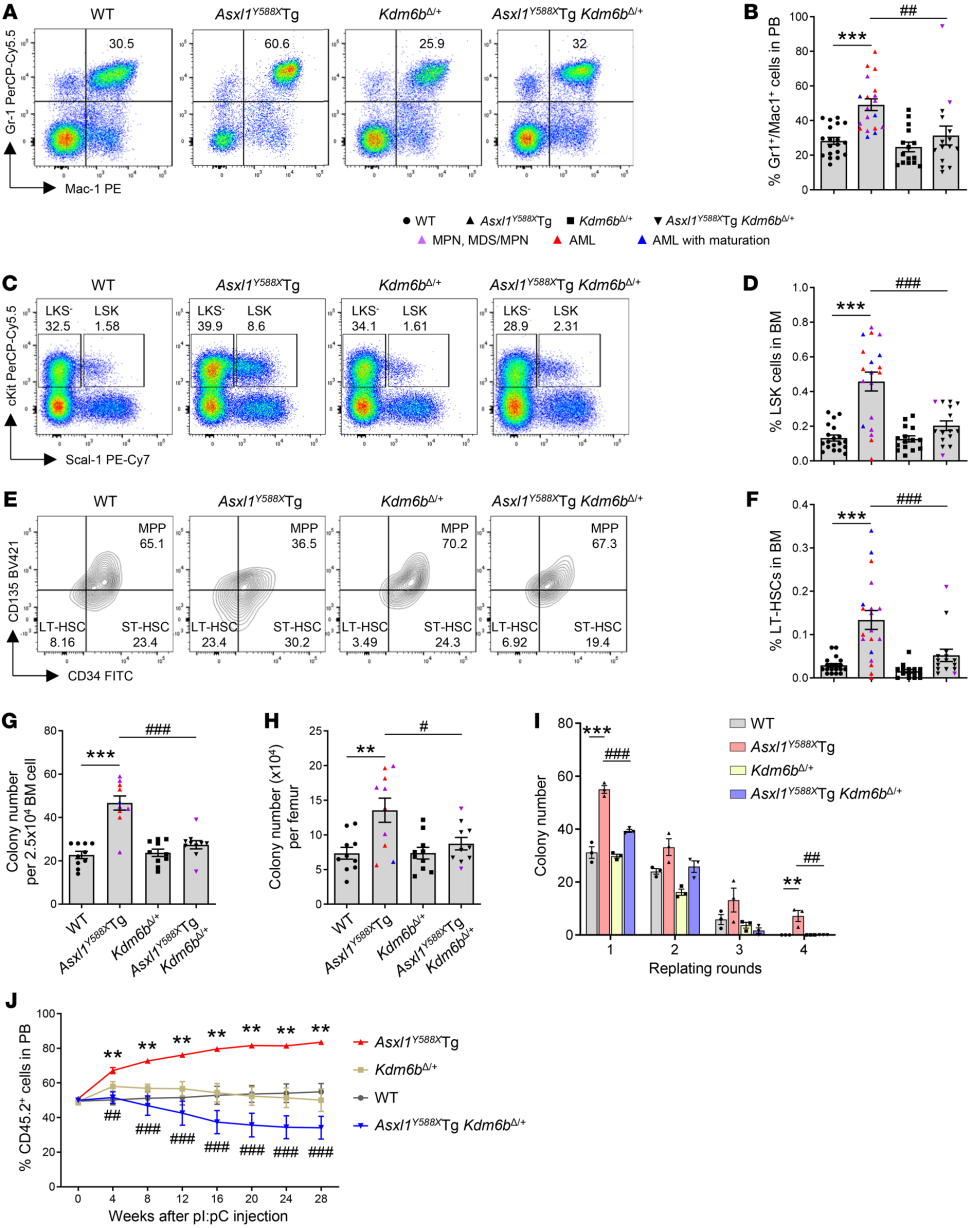
Heterozygous deletion of *Kdm6b* restores ASXL1^aa1–587^–mediated HSC phenotypes and myeloid differentiation. (**A**) Flow cytometric analysis of myeloid cells in PB from representative mice of each genotype. (**B**) The frequencies of Gr1^+^Mac1^+^ cells in PB from WT (*n* = 19), *Asxl1^Y588X^*Tg (*n* = 19), *Kdm6b^Δ/+^* (*n* = 15), and *Asxl1^Y588X^*Tg *Kdm6b^Δ/+^* (*n* = 15) mice. (**C** and **E**) Flow cytometric analysis of HSPCs in BM cells from representative mice of each genotype. (**D** and **F**) Quantification of the percentages of LSK cells (**D**) and LT-HSCs (**F**) in BM from WT (*n* = 19), *Asxl1^Y588X^*Tg (*n* = 19), *Kdm6b^Δ/+^* (*n* = 15), and *Asxl1^Y588X^*Tg *Kdm6b^Δ/+^* (*n* = 15) mice. (**G** and **H**) Colony-forming assay using BM cells from WT, *Asxl1^Y588X^*Tg, *Kdm6b^Δ/+^*, and *Asxl1^Y588X^*Tg *Kdm6b^Δ/+^* mice (*n* = 10 per genotype). (**I**) Serial cell replating assays using whole BM cells (*n* = 3 mice per genotype) were performed to determine HSC self-renewal capability. The cells were replated weekly for 4 weeks. (**J**) Percentages of donor-derived CD45.2^+^ cells in the PB of recipient animals at indicated time points (*n* = 5 per genotype). Data represent the mean ± SEM. **P < 0.01 and ***P < 0.001 vs. WT mice, and ^#^*P* < 0.05, ^##^*P* < 0.01, and ^###^*P* < 0.001 vs. *Asxl1^Y588X^*Tg mice, by 1-way ANOVA with Tukey’s multiple-comparison test (**B**, **D**, and **F**–**J**).

To determine whether heterozygous deletion of *Kdm6b* restores ASXL1^aa1–587^–mediated myeloid differentiation in vivo, we performed flow cytometric analysis on the PB of the 4 different genotypes of mice. Consistent with our previous findings, an increased proportion of myeloid cells (Gr1^+^Mac1^+^) was observed in the PB of *Asxl1^Y588X^*Tg mice compared with WT mice (Figure 3, A and B). In contrast, the frequency of the Gr1^+^Mac1^+^ myeloid population was significantly decreased in the PB of *Asxl1^Y588X^*Tg *Kdm6b^Δ/+^* mice compared with that in *Asxl1^Y588X^*Tg mice and indistinguishable from that in WT mice. The frequencies of B220^+^ cells and T cells in the PB were decreased in *Asxl1^Y588X^*Tg mice compared with WT mice, while their frequencies in *Asxl1^Y588X^*Tg *Kdm6b^Δ/+^* mice were relatively higher compared with those in *Asxl1^Y588X^*Tg mice and nearly approached those of WT mice (Supplemental Figure 3, A and B).

To assess whether *Kdm6b* loss affects ASXL1^aa1–587^–mediated increase of the HSC pool in vivo, we analyzed subpopulations of HSPCs in the BM cells by flow cytometry. No significant differences in the BM cellularity were observed among the 4 genotypes of mice (Supplemental Figure 3C). Flow cytometric analysis revealed that the percentages of Lin^−^Sca1^+^cKit^+^ (LSK) cells, long-term HSCs (LT-HSCs; LSKCD34^–^CD135^–^), and short-term HSCs (ST-HSCs; LSKCD34^+^CD135^–^) in the BM of *Asxl1^Y588X^*Tg mice were significantly higher than those in WT mice, and loss of 1 allele of *Kdm6b* significantly reduced the percentages of those cell populations in *Asxl1^Y588X^*Tg mice (Figure 3, C–F, and Supplemental Figure 3D). However, we did not observe any increase of LSK cells in the BM cells from young *Asxl1^Y588X^*Tg mice (Supplemental Figure 3, E and F). The frequency of Lin^−^cKit^+^Sca1^−^ (LKS^−^) cells in the BM was comparable among the 4 genotypes (Supplemental Figure 3, E–G). To examine the effects of *Kdm6b* deletion on BM HSPC functions in *Asxl1^Y588X^*Tg mice, we next performed CFU-C and serial replating assays. While the frequency of CFU-Cs was significantly higher in the BM of *Asxl1^Y588X^*Tg mice compared with WT mice, deletion of 1 allele of *Kdm6b* in *Asxl1^Y588X^*Tg mice restored the CFU-C frequency in the BM to that of WT (Figure 3, G and H, and Supplemental Figure 3H). In contrast to the increased replating potential of *Asxl1^Y588X^*Tg BM cells, we detected significantly lower replating activity over 4 successive replating periods in *Asxl1^Y588X^*Tg *Kdm6b^Δ/+^* cell cultures (Figure 3I), suggesting that *Kdm6b* deletion decreased the self-renewal capacity of *Asxl1^Y588X^*Tg cells.

To examine the repopulating capacity of *Asxl1^Y588X^*Tg *Kdm6b^Δ/+^* BM cells in vivo, we performed competitive transplantation assays by injecting equal numbers of BM cells from BoyJ mice (CD45.1^+^) and WT, *Mx1Cre*^+^ *Kdm6b^fl/+^*, *Asxl1^Y588X^*Tg, or *Asxl1^Y588X^*Tg *Mx1Cre^+^* *Kdm6b^fl/+^* mice (CD45.2^+^) into lethally irradiated BoyJ recipient mice (CD45.1^+^) (Supplemental Figure 3I). *Kdm6b* deletion was induced by pI:pC injection upon confirmation of comparable engraftment rates of CD45.1^+^ versus CD45.2^+^ cells in the PB of the recipient mice. In agreement with our previous reports (19), we observed that the recipient mice with transplanted *Asxl1^Y588X^*Tg BM cells had a higher percentage of CD45.2^+^ cells in their PB than the recipients with transplanted WT cells (Figure 3J). In contrast, the recipients receiving *Asxl1^Y588X^*Tg *Kdm6b^Δ/+^* BM cells had significantly lower levels of CD45.2^+^ cell population compared with the mice with transplanted *Asxl1^Y588X^*Tg cells (Figure 3J and Supplemental Figure 3J). These results indicate that decreased expression of *Kdm6b* in *Asxl1^Y588X^*Tg cells rescues the ASXL1^aa1–587^-mediated abnormal HSPC phenotypes in vivo.

### Heterozygous deletion of Kdm6b decreases ASXL1^aa1–587^–mediated transcription activation in HSPCs.

**Figure 4:**
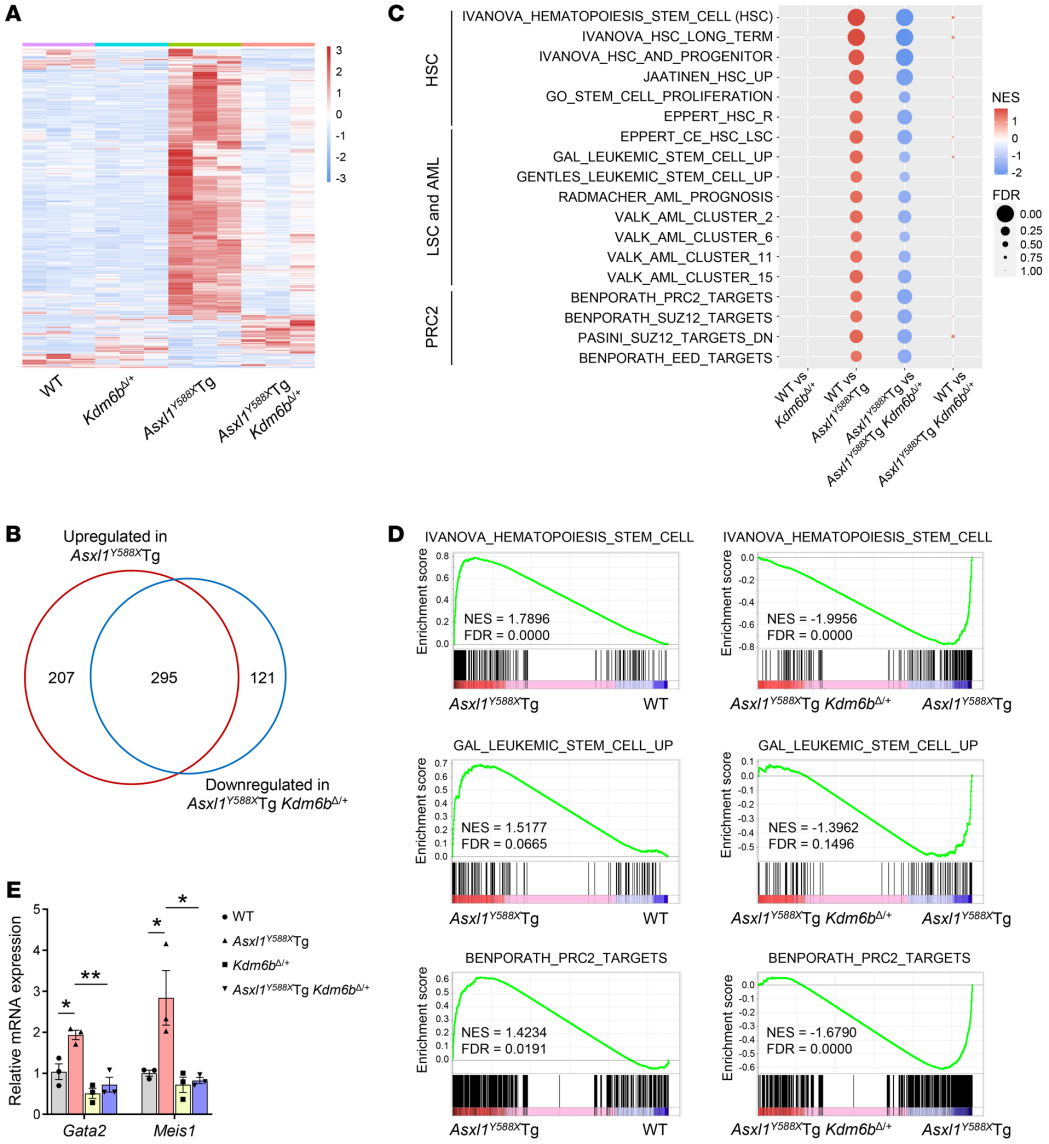
Heterozygous deletion of *Kdm6b* decreases ASXL1^aa1–587^–mediated transcription activation in HSPCs. (**A**) Heatmap displaying gene expression for all genes differentially expressed in LK cells from each mutant genotype relative to WT controls (FDR < 0.05 and |fold change| > 1.5, *n* = 3 mice per genotype). (**B**) Venn diagram showing the overlap of dysregulated genes in *Asxl1^Y588X^*Tg LK cells (compared with WT cells) and *Asxl1^Y588X^*Tg *Kdm6b^Δ/+^* cells (compared with *Asxl1^Y588X^*Tg cells). (**C**) GSEA with NES and FDR values for gene sets of HSC, LSC and AML, and PRC2 in *Asxl1^Y588X^*Tg LK cells. The colors reflect scaled NES, representing the degree of expression change. Sizes of circles represent the FDR value. (**D**) GSEA plots show that genes involved in the regulation of HSC, LSC, and PRC2 targets are upregulated in *Asxl1^Y588X^*Tg LK cells, but downregulated in *Asxl1^Y588X^*Tg *Kdm6b^Δ/+^* cells. NES, *P* value, and FDR are shown. (**E**) qPCR verified the change in mRNA levels of *Gata2* and *Meis1* (*n* = 3 mice per genotype). Data represent the mean ± SEM. *P < 0.05 and **P < 0.01, by 1-way ANOVA with Tukey’s multiple-comparison test.

To determine whether deletion of *Kdm6b* could rescue the abnormal molecular pathways caused by ASXL1^aa1–587^ expression in HSPCs, we examined the gene expression profiles of WT, *Kdm6b^Δ/+^*, *Asxl1^Y588X^*Tg, and *Asxl1^Y588X^*Tg *Kdm6b^Δ/+^* Lin^–^cKit^+^ (LK) cells by RNA-Seq. Compared with WT cells, *Asxl1^Y588X^*Tg cells had an aberrant gene expression signature consisting of 502 upregulated and 20 downregulated genes (fold change > 1.5 and FDR < 0.05; Figure 4A and Supplemental Figure 4A). The functional enrichment analysis with 522 dysregulated genes in *Asxl1^Y588X^*Tg cells revealed that 109 genes were involved in HSC functions, leukemic stem cells (LSCs), and AML pathways, including *Meis1*, *Prdm16*, *Gata2*, and *Hoxb5*, which are known to be key for HSPC function and leukemogenesis (Supplemental Table 3). Notably, we observed a restoration of *ASXL1^aa1–587^–*associated dysregulated genes in *Asxl1^Y588X^*Tg *Kdm6b^Δ/+^* cells (Figure 4A). A substantial proportion of upregulated genes (295/502, 58.8%) in *Asxl1^Y588X^*Tg cells was restored in *Asxl1^Y588X^*Tg *Kdm6b^Δ/+^* cells to WT cell level (Figure 4B), suggesting that KDM6B may function as a main downstream regulator of truncated ASXL1 in HSPCs. GSEA showed that the upregulated genes in *Asxl1^Y588X^*Tg cells were enriched for HSCs, LSCs, and PRC2 targets (Figure 4, C and D, and Supplemental Figure 4B). In contrast, the transcriptional activity of those PRC2 target genes was repressed in *Asxl1^Y588X^*Tg *Kdm6b^Δ/+^* cells (Figure 4, C and D, and Supplemental Figure 4B), whereas the mRNA levels of PRC2 subunits, including *Ezh1*, *Ezh2*, *Suz12*, and *Eed*, were comparable among the 4 genotypes of cells (Supplemental Figure 4C). Furthermore, HSC- and LSC-associated genes in *Asxl1^Y588X^*Tg *Kdm6b^Δ/+^* HSPCs were also restored to WT levels (Figure 4, C and D). qPCR confirmed the changes in selected genes *Gata2* and *Meis1*, which are associated with HSPC functions and myeloid differentiation (Figure 4E and Supplemental Figure 4D). However, we did not observe these changes in young mice (Supplemental Figure 4, E and F).

### Genetic reduction of Kdm6b in Asxl1^Y588X^Tg mice normalizes the levels of dysregulated genes by restoring H3K27me3 levels.

**Figure 5:**
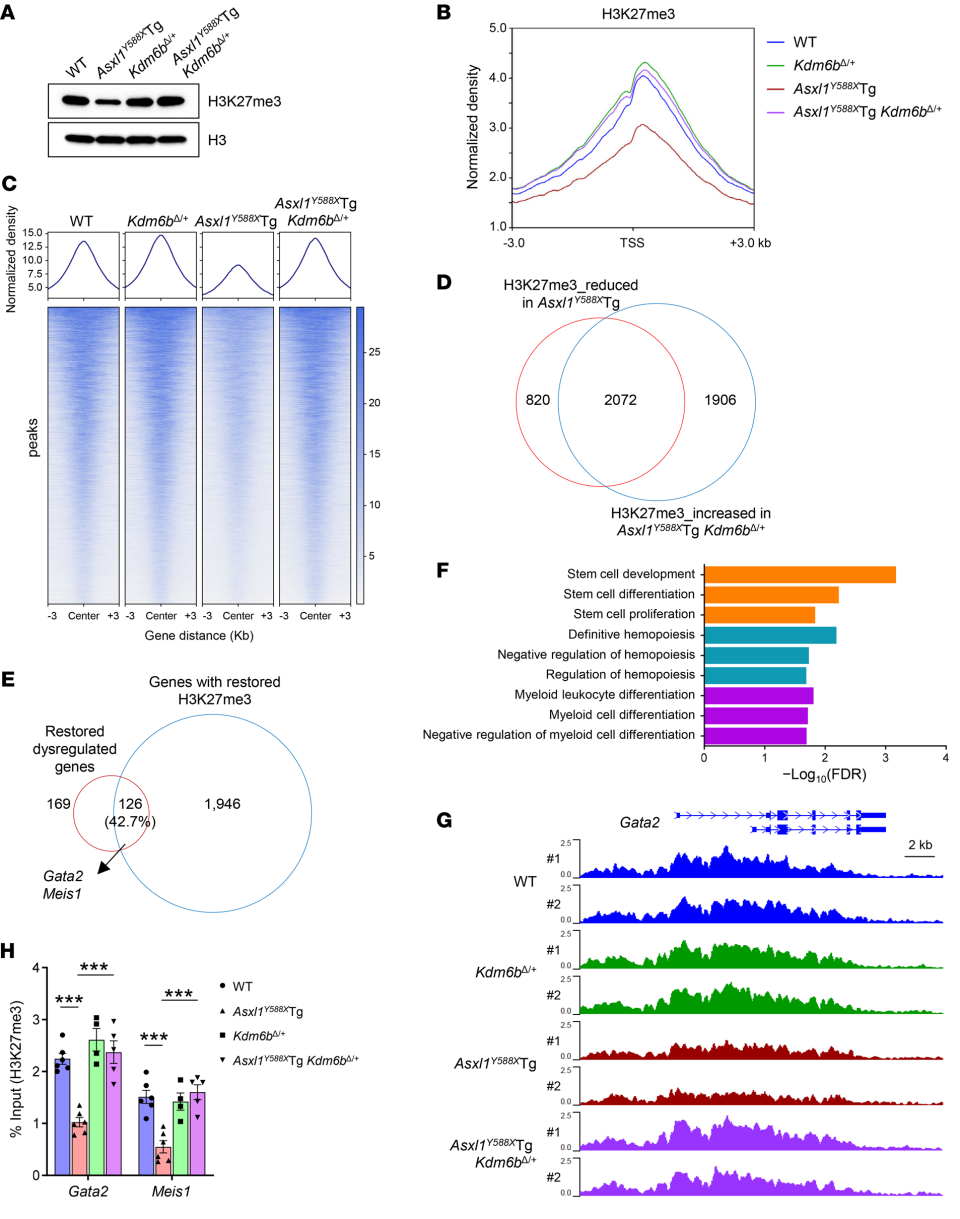
Genetic reduction of *Kdm6b* in *Asxl1^Y588X^*Tg mice normalizes the levels of dysregulated genes by restoring H3K27me3 levels. (**A**) Western blot showing the levels of H3K27me3 in BM cells from representative mice of each genotype. (**B**) Global levels of H3K27me3 at transcription start site (TSS) and 3-kb regions surrounding TSS. The coverages were normalized by the sequencing depth and averaged in 2 biological replicates. (**C**) Heatmaps of normalized H3K27me3 ChIP-Seq read densities centered on the midpoints of 6,305 H3K27me3-changed regions (FDR < 0.05). Each row represents a single region. (**D**) Venn diagram showing the overlap of genes between reduced H3K27me3 in *Asxl1^Y588X^*Tg (compared with WT controls) and increased H3K27me3 in *Asxl1^Y588X^*Tg *Kdm6b^Δ/+^* LK cells (compared with *Asxl1^Y588X^*Tg). The number of genes in each section of the diagram is shown. (**E**) Venn diagram showing the overlap between genes with restored H3K27me3 (**D**) and restored dysregulated genes (in Figure 4B) in LK cells. The number of genes in each section of the diagram is shown. (**F**) Functional enrichment analysis for 126 overlapping genes in **E**. Representative significantly enriched pathways are displayed (FDR < 0.05). (**G**) Normalized H3K27me3 signals on the *Gata2* gene loci are shown. (**H**) ChIP-qPCR verified the reduction of H3K27me3 occupancies at the promoter regions of *Gata2* and *Meis1* genes (*n* = 4–6 mice per genotype). Data represent the mean ± SEM. ***P < 0.001, by 1-way ANOVA with Tukey’s multiple-comparison test.

KDM6B is an H3K27-specific demethylase that catalyzes the transition of H3K27me3 and H3K27me2 to H3K27me1 on bulk histone substrates, with H3K27me3 being a preferred substrate (27). To determine whether *Kdm6b* loss in *Asxl1^Y588X^*Tg cells affects the global levels of H3K27me3, we performed Western blot analyses using BM cells of the 4 different genotypes of mice. Compared with *Asxl1^Y588X^*Tg cells, which showed a much lower level of H3K27me3, the H3K27me3 level in *Asxl1^Y588X^*Tg *Kdm6b^Δ/+^* BM cells was restored to that of WT cells (Figure 5A and Supplemental Figure 5A). To evaluate whether the dysregulated gene expression was associated with the changes of H3K27me3 occupancies in HSPCs, we performed H3K27me3 ChIP assays followed by sequencing using LK cells from WT, *Asxl1^Y588X^*Tg, *Kdm6b^Δ/+^*, and *Asxl1^Y588X^*Tg *Kdm6b^Δ/+^* mice. A substantial reduction in genome-wide H3K27me3 occupancy was observed in *Asxl1^Y588X^*Tg cells compared with WT controls (*P* < 1.4 × 10^−38^, unpaired *t* test; Figure 5B and Supplemental Figure 5B). While the H3K27me3 levels were slightly increased in *Kdm6b^Δ/+^* cells, heterozygous deletion of *Kdm6b* restored the reduced levels of H3K27me3 in *Asxl1^Y588X^*Tg cells to the same levels as those of WT (Figure 5, B and C, and Supplemental Figure 5B). Specifically, H3K27me3 levels of 2,072 (71.6%) genes that were decreased in *Asxl1^Y588X^*Tg cells compared with WT cells were restored in *Asxl1^Y588X^*Tg *Kdm6b^Δ/+^* cells to WT levels (Figure 5D). Additionally, the changes in H3K27me3 peaks between *Asxl1^Y588X^*Tg and *Asxl1^Y588X^*Tg *Kdm6b^Δ/+^* were mainly located at the promoter regions (Supplemental Figure 5, C and D), suggesting a responsible role of *Kdm6b* loss–mediated H3K27me3 restoration for the normalization of gene expression in *Asxl1^Y588X^*Tg *Kdm6b^Δ/+^* HSPCs.

To determine the impact of decreased H3K27me3 levels on gene expression in *Asxl1^Y588X^*Tg LK cells, we integrated the RNA-Seq and ChIP-Seq data sets. While 240 (47.8%) upregulated genes were associated with reduced H3K27me3 peaks in *Asxl1^Y588X^*Tg mice (Supplemental Figure 5E), 245 (58.9%) of the downregulated genes in *Asxl1^Y588X^*Tg *Kdm6b^Δ/+^* cells were overlapped with the increased H3K27me3 peaks compared with *Asxl1^Y588X^*Tg cells (Supplemental Figure 5F). We further determined whether the overlapped dysregulated genes were associated with the changes of H3K27me3 levels and found that 126 (42.7%) dysregulated genes were associated with H3K27me3 changes in *Asxl1^Y588X^*Tg *Kdm6b^Δ/+^* cells (Figure 5E). Gene Ontology analyses revealed that these overlapped dysregulated genes in *Asxl1^Y588X^*Tg *Kdm6b^Δ/+^* cells were enriched in hematopoiesis, stem cell function, and myeloid differentiation (Figure 5F). We also examined the occupancies of H3K27me3 on genes associated with HSC function and myeloid differentiation, including *Gata2* and *Meis1*. The gene changes in *Asxl1^Y588X^*Tg and *Asxl1^Y588X^*Tg *Kdm6b^Δ/+^* cells were correlated with the changes of H3K27me3 (Figure 5G and Supplemental Figure 5G). ChIP-qPCR further confirmed the changes in H3K27me3 at the promoters of *Gata2* and *Meis1* genes (Figure 5H). These data suggest that the key upregulated genes are directly related to the levels of H3K27me3 in *Asxl1^Y588X^*Tg HSPCs, and that genetic reduction of *Kdm6b* restored H3K27me3 levels and correlatively normalized the expression of genes critical for HSC function and myeloid differentiation. Together, these results suggest that the antileukemia effect of genetic reduction of *Kdm6b* in *Asxl1^Y588X^*Tg mice is due to the restoration of H3K27me3 and corresponding leukemogenic gene suppression.

### Pharmacologic KDM6B inhibition blocks the growth of ASXL1-mutated leukemia cells.

**Figure 6:**
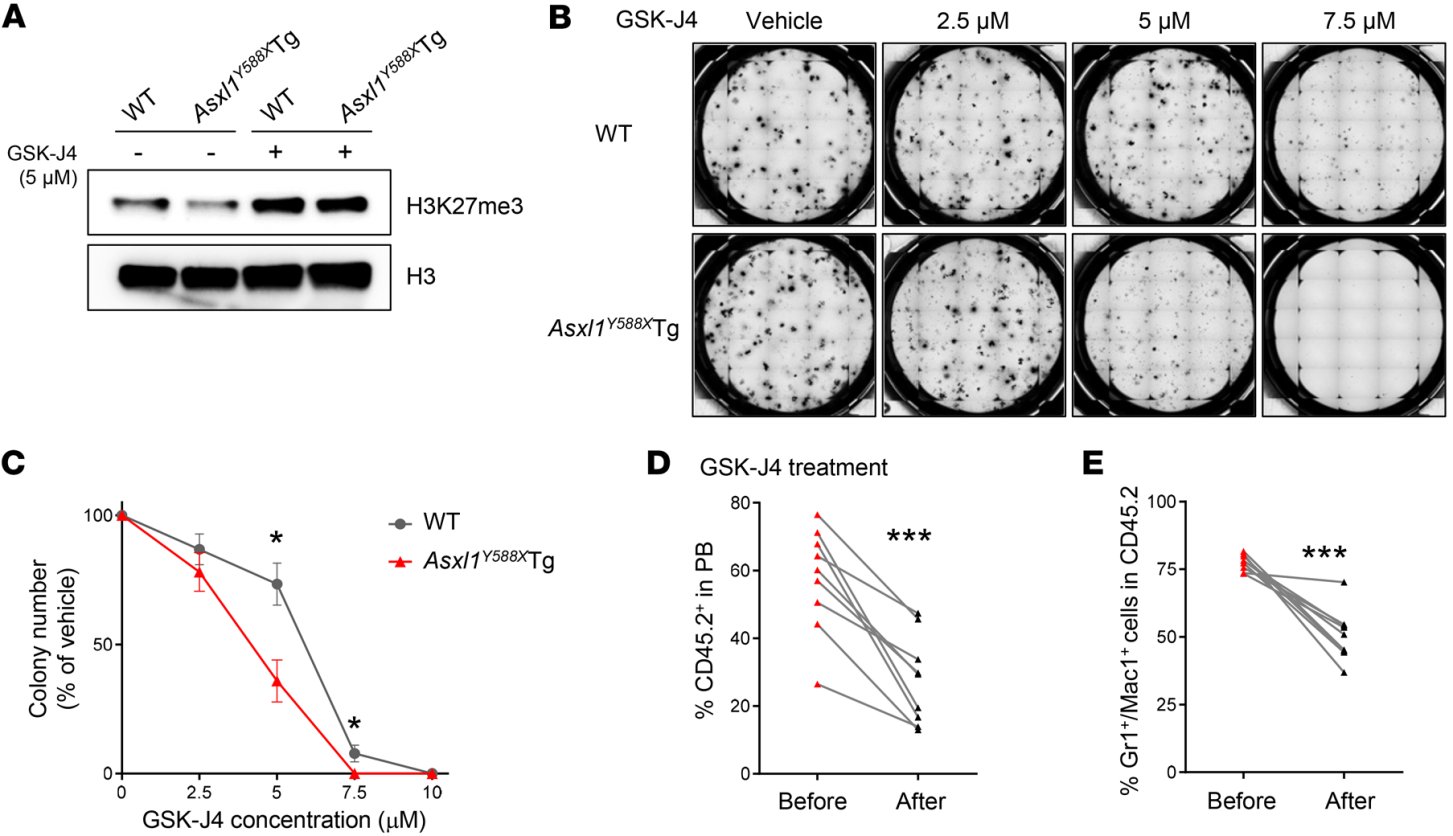
*Asxl1^Y588X^*Tg BM cells are sensitive to the KDM6B inhibitor GSK-J4. (**A**) Western blot showing the levels of H3K27me3 in BM cells treated with 5 μM GSK-J4 for 24 hours. (**B** and **C**) Colony-forming assays using BM cells with or without GSK-J4 treatment (*n* = 5 mice per genotype). Representative images of colony formation from each condition are shown. The concentration of GSK-J4 is indicated. WT, IC_50_ = 5.63 μM; *Asxl1^Y588X^*Tg, IC_50_ = 3.86 μM. (**D** and **E**) The frequencies of CD45.2^+^ (**D**) and Gr1^+^Mac1^+^ cells (**E**) in PB from *Asxl1^Y588X^*Tg mice treated with GSK-J4 (*n* = 9). Data represent the mean ± SEM. *P < 0.05 and ***P < 0.001, by unpaired Student’s *t* test (**C**) and paired Student’s *t* test (**D** and **E**).

GSK-J4 is an H3K27me3 demethylase inhibitor that influences gene transcription by increasing the levels of H3K27me3 at gene promoter regions (44). We examined H3K27me3 levels in WT and *Asxl1^Y588X^*Tg BM cells treated with GSK-J4. As expected, GSK-J4 restored H3K27me3 levels in *Asxl1^Y588X^*Tg BM cells (Figure 6A and Supplemental Figure 6A). To assess the effect of GSK-J4 on *Asxl1^Y588X^*Tg HSPC functions in vitro, we conducted semisolid methylcellulose culture to determine the frequency of CFU-Cs in the BM of these mice. GSK-J4 preferentially inhibited CFU-C formation from *Asxl1^Y588X^*Tg BM cells compared with WT cells (Figure 6, B and C). In addition, GSK-J4 decreased the mRNA levels of *Gata2* and *Meis1* in *Asxl1^Y588X^*Tg cells (Supplemental Figure 6B).

To determine the therapeutic effects of GSK-J4 on the hematopoietic phenotypes of *Asxl1^Y588X^*Tg mice in vivo, we performed tumor transfer assays by injecting *Asxl1^Y588X^*Tg leukemic cells into WT recipients, and 2 weeks after the transplantation, the recipients were treated with GSK-J4 or vehicle control for 5 weeks (Supplemental Figure 6C). PB bleeding and subsequent flow cytometric analysis revealed a significant inhibition of leukemic cell engraftment in GSK-J4–treated mice (Figure 6, D and E, and Supplemental Figure 6D). Further histologic analysis of the BM cell cytospin preparations of these recipient mice demonstrated a reduction in blasts in the GSK-J4–treated recipients reconstituted with *Asxl1^Y588X^*Tg leukemic cells compared with vehicle control recipient mice (Supplemental Figure 6E).

**Figure 7:**
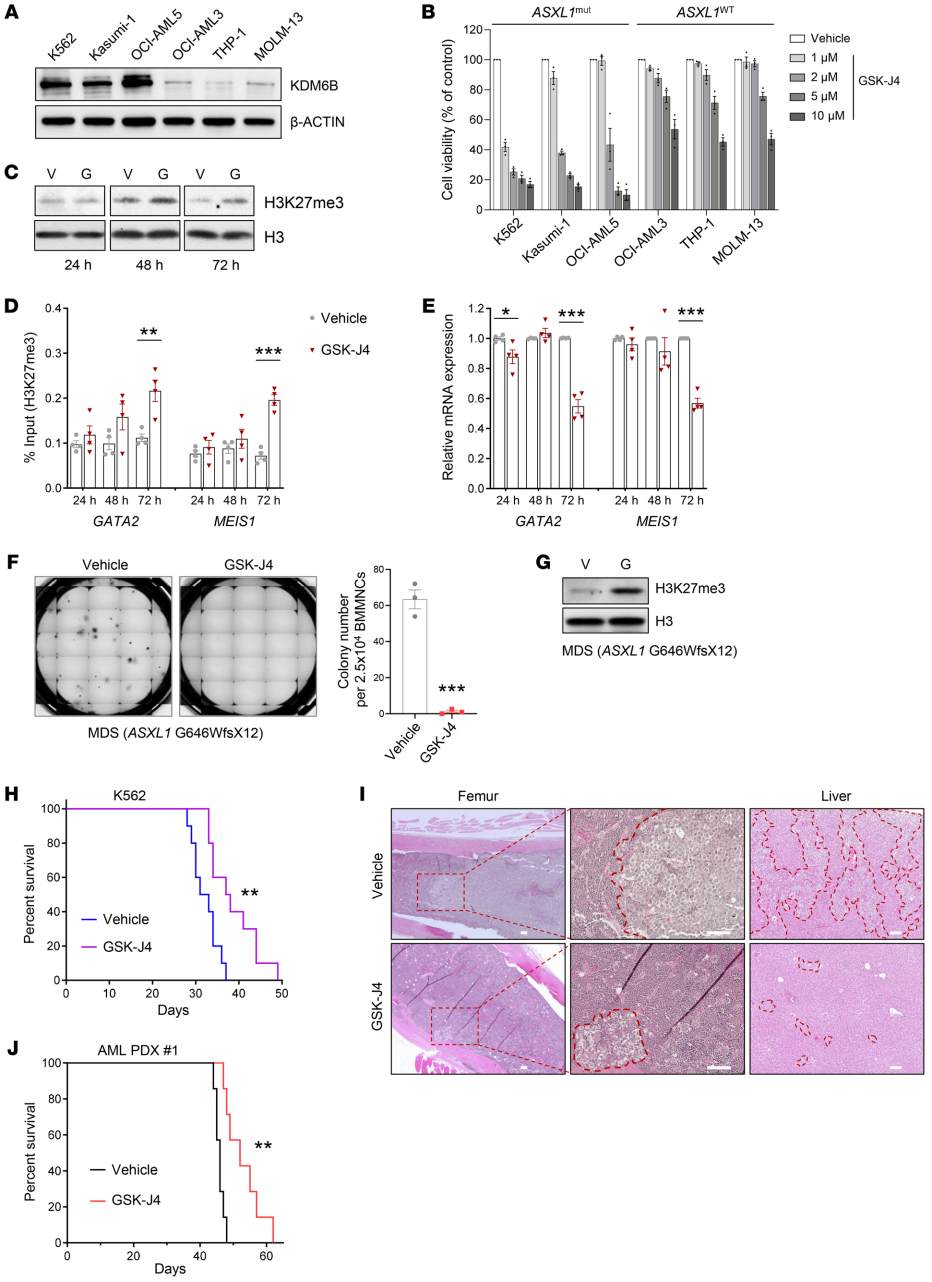
Pharmacologic KDM6B inhibition blocks the growth of *ASXL1* mutation–mediated leukemic cells. (**A**) Western blot showing the protein level of KDM6B in leukemia cell lines. (**B**) Human leukemia cells were treated with various concentrations of GSK-J4 for 72 hours. The viabilities of the cultured cells were measured using CellTiter-Glo luminescent assay. (**C**) Western blot analysis of H3K27me3 levels in OCI-AML5 cells treated with vehicle (V) or 5 μM GSK-J4 (G) after 24, 48, and 72 hours. (**D**) ChIP-qPCR showing the levels of H3K27me3 occupancies at the promoter regions of *GATA2* and *MEIS1*. (**E**) The relative mRNA levels of *GATA2* and *MEIS1* in OCI-AML5 cells were analyzed by qPCR. (**F**) Colony-forming assay using primary BM mononuclear cells (BMMNCs) from an MDS patient (*ASXL1* G646WfsX12) with or without GSK-J4 treatment. Representative images of colony formation are shown. The images were taken on the 14th day of the assay. (**G**) Western blot showing the levels of H3K27me3 in primary mononuclear cells from an MDS patient with the treatment of 5 μM GSK-J4 for 72 hours. (**H**) Kaplan-Meier survival curve representing the survival of K562-transplanted NSG mice treated with DMSO or 50 mg/kg GSK-J4 (*n* =10 mice per group). (**I**) Representative H&E staining of femur and liver sections from the mice in **H**. Scale bars: 100 μm. (**J**) Kaplan-Meier survival curve for AML PDX #1–transplanted NSG mice treated with DMSO or 50 mg/kg GSK-J4 (*n* = 7 mice per group). Data were derived from 3–4 independent experiments and represent the mean ± SEM. *P < 0.05, **P < 0.01, and ***P < 0.001, by unpaired Student’s *t* test (**D**–**F**) or log-rank (Mantel-Cox) test (**H** and **J**).

To validate our findings in mice, we applied GSK-J4 to the culture of K562 cells, a human leukemic cell line with high KDM6B level, and assessed its impact on cell proliferation. GSK-J4 dramatically inhibited the growth of K562 cells in liquid cultures in a time-dependent manner (Supplemental Figure 7A). We also examined the impact of GSK-J4 on the cell growth of several other leukemia cell lines with lower expression of KDM6B, including OCI-AML3, THP-1, and MOLM-13 cells (Figure 7A). Treatment of KDM6B-high leukemic cells with GSK-J4 dramatically reduced the cell viability in a dose-dependent manner, whereas minimal effect was seen in KDM6B-low leukemic cells. The IC_50_ values of GSK-J4 in KDM6B-high leukemic cells were lower than those in KDM6B-low cells (Figure 7B and Supplemental Figure 7B). Interestingly, all the cell lines harboring *ASXL1* truncating mutations were sensitive to GSK-J4 treatment, including K562 (Y591X), Kasumi-1 (G646WfsX12), and OCI-AML5 (Y591X) cells. In line with these results, GSK-J4 treatment increased H3K27me3 levels in OCI-AML5 cells, leading to decreased expression of *GATA2* and *MEIS1* (Figure 7, C–E). In contrast, GSK-J4 had only minimal effects on the levels of H3K27me3, *GATA2*, and *MEIS1* in THP-1 cells (Supplemental Figure 7, C–E). In addition, the presence of GSK-J4 markedly decreased the frequencies of CFU-Cs derived from K562, Kasumi-1, and OCI-AML5 cells compared with vehicle control, but did not impact on the CFU-Cs of THP-1 cells (Supplemental Figure 7, F and G). Importantly, the addition of GSK-J4 significantly inhibited colony formation from primary cells of myeloid malignancy patients with *ASXL1* mutations (Figure 7F and Supplemental Figure 7H), whereas a minimal effect of GSK-J4 was observed in the primary cells of a myeloid malignancy patient without *ASXL1* mutation (Supplemental Figure 7I). Moreover, GSK-J4 robustly increased the levels of H3K27me3 in primary patient cells with *ASXL1* mutations (Figure 7G and Supplemental Figure 7, J and K).

We further created a leukemic xenograft mouse model by transplanting K562 cells into sublethally irradiated NSG mice (45) and assessed the effects of GSK-J4 on disease burden (Supplemental Figure 7L). GSK-J4 significantly extended the survival of NSG mice xenografted with K562 cells compared with vehicle control (Figure 7H). Morphologic analysis of BM cytospins and histologic analysis of the femur and liver demonstrated a decreased leukemia burden in the femur and liver of GSK-J4–treated mice compared with the vehicle controls (Figure 7I and Supplemental Figure 7M). In addition, we found that GSK-J4 treatment led to significantly prolonged survival and reduced leukemia burden in mice xenografted with AML patient-derived xenograft (PDX) #1 cells (*ASXL1* G646WfsX12) without reducing body weight (Figure 7J and Supplemental Figure 7, N–Q). In contrast, GSK-J4 treatment did not improve survival nor reduce disease burden in NSG mice xenografted with THP-1 or AML PDX #2 (*ASXL1*-WT) cells (Supplemental Figure 7, R–T). These findings indicate that the overexpression of KDM6B associated with *ASXL1* mutation in human leukemia cells renders them highly vulnerable to targeted inhibition of KDM6B, and provide solid preclinical evidence for the potential of pharmacologic inhibition of KDM6B for the treatment of *ASXL1* mutation–associated myeloid malignancies.

Finally, since *GATA2* and *MEIS1* have been shown to be important for HSPC function and leukemia transformation (46, 47), and their expression is upregulated in *Asxl1^Y588X^*Tg cells (Figure 4E) and can be downregulated with KDM6B inhibition, we performed shRNA knockdown of *GATA2* and *MEIS1* in K562 and Kasumi-1 cells and examined the CFU-C frequency to establish their roles in *ASXL1* mutation–mediated leukemia transformation. Knockdown of *GATA2* or *MEIS1* with 2 different shRNAs resulted in a significant reduction in the frequency of CFU-Cs in K562 cells as well as Kasumi-1 cells compared with cells transduced with a control shRNA (Supplemental Figure 7, U–X). These results indicate that GATA2 and MEIS1 are crucial downstream factors of leukemogenicity associated with *ASXL1* truncating mutation that can be targeted either directly or indirectly via KDM6B inhibition to improve HSPC function and prevent leukemia transformation.

## Discussion

Herein we employed genetic and pharmacologic approaches to evaluate the therapeutic potential of targeting KDM6B in *ASXL1* mutation–associated myeloid malignancies. We have demonstrated that *Kdm6b* genetic deletion or pharmacologic inhibition of KDM6B corrects the abnormal HSPC behavior and prevents myeloid malignancy development caused by *ASXL1^Y591X^* (*Asxl1^Y588X^* in mice) mutation.

ASXL1 loss or mutation impairs the recruitment of PRC2 complex proteins to chromatin, reducing H3K27me3 deposition, and leading to associated leukemogenic gene dysregulation and the development of myeloid malignancies (16–18, 22, 48). The appropriate levels of H3K27me3 are tightly maintained by the histone methyltransferase EZH2, a component of the PRC2 complex, and the demethylases KDM6A and KDM6B. Currently, to our knowledge, there are limited strategies for targeting PRC2 proteins to modulate the deposition of H3K27me3 at target loci in the context of *ASXL1* alterations, given the critical importance of ASXL1 for the recruitment of PRC2 complex to chromatin. Therefore, manipulation of KDM6B becomes the only viable strategy for increasing the levels of H3K27 methylation in *ASXL1*-mutant cells. *Kdm6b* loss in mice has been shown to impair HSC self-renewal and enhance HSC differentiation (33), and *Kdm6a*-deficient mice have been shown to exhibit abnormal differentiation with myeloid skewing, impaired hematopoietic reconstituting ability, and increased susceptibility to leukemia (49). On the other hand, overexpression of KDM6B in the murine hematopoietic compartment also leads to impairment of BM HSPCs and ineffective hematopoiesis (50). These studies underscore the importance of maintaining KDM6B levels for normal HSC function and hematopoiesis. We found that the expression of KDM6B is increased in *Asxl1^Y588X^*Tg cells compared with WT controls, while KDM6A levels between the two are similar. Importantly, inhibition of KDM6B restored H3K27me3 levels in *Asxl1^Y588X^*Tg *Kdm6b^Δ/+^* BM cells, prompting us to initiate preclinical studies to determine whether targeting KDM6B could potentially benefit patients with *ASXL1* mutation–associated myeloid malignancies. We showed that loss of KDM6B decreased the self-renewal capacity of *Asxl1^Y588X^*Tg HSCs and K562 leukemic cells, which was accompanied by the restoration of global H3K27me3 levels. The data we have presented herein demonstrate that heterozygous deletion of *Kdm6b* blocks the development of *ASXL1* mutation–mediated myeloid malignancies, and that the KDM6B inhibitor GSK-J4 likewise attenuates *ASXL1*-mutated leukemic cell growth and tumor burden in preclinical mouse models. Future studies that aim to decipher the mechanism by which *ASXL1* mutation leads to the dysregulation of KDM6B levels in leukemic cells may reveal additional strategies for restoring H3K27me3 levels.

The repressive histone mark H3K27me3 is highly correlated with silent loci and is important for regulating developmental and oncogenic genes (51). Consistently, our RNA-Seq and ChIP-Seq analyses showed that almost half (47.8%) of upregulated genes in *Asxl1^Y588X^*Tg HSPCs were correlated with reduced H3K27me3 occupancy. Notably, genetic reduction of *Kdm6b* in *Asxl1^Y588X^*Tg HSPCs normalized a substantial proportion (58.9%) of upregulated genes by restoring H3K27me3 levels. *Gata2* and *Meis1*, genes that are crucial for HSPC function and myeloid differentiation, were upregulated in *Asxl1^Y588X^*Tg HSPCs, and downregulated with genetic reduction of *Kdm6b* and inhibition of KDM6B with GSK-J4. ChIP-qPCR confirmed the increased H3K27me3 occupancy at the promoter regions of *Gata2* and *Meis1*, suggesting a tight correlation between H3K27me3 and gene expression in *Asxl1^Y588X^*Tg HSPCs. Moreover, *GATA2* and *MEIS1* knockdown in the leukemic cell lines K562 and Kasumi-1 reduced the frequency of CFU-Cs, verifying that *GATA2* and *MEIS1* could be potential therapeutic targets for *ASXL1* mutation–associated myeloid malignancies.

GSK-J4 has been suggested as a potential clinical agent for hematologic malignancies (35, 38). While GSK-J4 was originally described as a specific inhibitor for KDM6A and KDM6B, further studies found that GSK-J4 has broad activities in inhibiting Jumonji C domain–containing lysine demethylases with differential preference (52). Thus, the development and preclinical testing of KDM6B inhibitors with greater specificity are warranted. Furthermore, since BET bromodomain inhibitors have also been shown to reduce the frequency of CFU-Cs in *Asxl1^Y588X^*Tg BM cells (19), the combinatory effect of KDM6B inhibitor with other targeted therapeutics (e.g., BET inhibitor) could potentially deepen treatment responses and further improve the survival of patients with *ASXL1*-mutated myeloid malignancies.

In summary, our data indicate that genetic reduction of *Kdm6b* alleviates the hematopoietic phenotypes and normalizes H3K27me3 levels in *Asxl1^Y588X^*Tg mice. Our findings further support the notion that insufficient H3K27 methylation due to truncating *Asxl1* mutation plays a central role in the initiation and progression of myeloid malignancies. Additionally, we have demonstrated that pharmacologic inhibition of KDM6B in *Asxl1*-mutant HSPCs restores H3K27me3 level, normalizes leukemogenic gene expression and HSPC function, and reduces the tumor burden in mouse models of *ASXL1* mutation–mediated leukemia. Importantly, our study provides scientific evidence for the therapeutic potential of targeting KDM6B in treating *ASXL1* mutation–associated myeloid malignancies.

## Methods

### Mouse models.

The generation of *Asxl1^Y588X^*Tg and *Kdm6b^fl/fl^* mice has been previously described (19, 43). *Mx1Cre* transgenic, BoyJ (CD45.1^+^), and NSG mice were purchased from The Jackson Laboratory. *Mx1Cre*-induced gene deletion was done by intraperitoneal injection of pI:pC (10 mg/kg; InvivoGen) 3 times every other day. The genotyping PCR primers are listed in Supplemental Table 4.

### Cell culture.

The human leukemia cell lines were obtained from the American Type Culture Collection or the Leibniz Institute DSMZ (Braunschweig, Germany). Human BM CD34^+^ cells were purchased from Lonza. K562, THP-1, and MOLM-13 cells were cultured in RPMI 1640 medium with 10% FBS and 1% penicillin-streptomycin. Kasumi-1 cells were cultured in RPMI 1640 medium with 20% FBS and 1% penicillin-streptomycin. OCI-AML5 and OCI-AML3 cells were cultured in α-MEM with 20% FBS and 1% penicillin-streptomycin. OCI-AML5 cultures were also supplemented with 10 ng/mL of human GM-CSF.

### Primary patient cells.

Deidentified human specimens were used, and cytogenetic information of myeloid malignancy patient cells is available in Supplemental Table 5. Mononuclear cells were isolated by Histopaque-1077 (MilliporeSigma) density centrifugation and cultured in RPMI 1640 medium supplemented with 10% FBS; 1% penicillin-streptomycin; human stem cell factor (hSCF; 100 ng/mL), thrombopoietin (hTPO; 100 ng/mL), interleukin-3 (hIL-3; 100 ng/mL), interleukin-6 (hIL-6; 100 ng/mL), and Fms-like tyrosine kinase 3 ligand (hFlt-3L; 100 ng/mL); and 0.75 μM StemRegenin 1.

### Plasmid constructs and transfection/nucleofection.

The all-in-one expression vector LentiCRISPRv2GFP, which contains Cas9 and human *KDM6B* sgRNA, was purchased from Synbio Technologies. The empty vector without sgRNA (Cas9 only) was used as control. CRISPR sgRNA sequence used was AACGGAACTATGGAGCCAAG. The shRNA expression vectors were purchased from VectorBuilder. shRNA sequences used were CTACAAGCTGCACAATGTTAACTCGAGTTAACATTGTGCAGCTTGTAG (shGATA2 #1); CCGGCACCTGTTGTGCAAATTCTCGAGAATTTGCACAACAGGTGCCGG (shGATA2 #2); TAGAGAAGGTACACGAATTATCTCGAGATAATTCGTGTACCTTCTCTA (shMEIS1 #1); and CAGAAGCCTCCTTACATTAAACTCGAGTTTAATGTAAGGAGGCTTCTG (shMEIS1 #2).

Nucleofection of K562 cells was performed according to the manufacturer’s instructions (SF Cell Line 4D-Nucleofector X Kit L, Lonza). Briefly, 1 × 10^6^ K562 cells were resuspended in 100 μL SF 4D-Nucleofector X solution. Cells were mixed with CRISPR vector and then transferred to nucleocuvette. Cells were electroporated with program FF-120 using a 4D nucleofector (4D-Nucleofector X Unit, Lonza). After nucleofection, prewarmed medium was used to transfer transfected cells in 12-well plates. Forty-eight hours after nucleofection, GFP^+^ cells were sorted into 96-well plates at 1 cell per well. Single-cell clones were verified by Western blot analysis, PCR, and Sanger sequencing.

293TN cells (System Biosciences) were cultured in DMEM supplemented with 10% FBS and transfected with different shRNA expression vectors using Lipofectamine 3000 (Thermo Fisher Scientific). The viral supernatant was harvested 48 hours after transfection. K562 and Kasumi-1 cells were transduced with the packaged viruses, and the positive cells were then sorted for further experiments using a BD FACSAria machine.

### Phenotypic analyses of mice.

The analyses were performed when *Asxl1^Y588X^*Tg or *Asxl1^Y588X^*Tg *Kdm6b^Δ/+^* mice became moribund (12–26 months; Supplemental Tables 1 and 2), and age-matched mice of the other genotypes were used as controls. PB was collected by tail vein bleeding and subjected to an automated blood count (Element HT5, Heska). For morphologic and lineage differential analysis, PB smears were subjected to May-Grünwald-Giemsa staining. Morphologic analyses of BM cells were performed on cytospins followed by May-Grünwald-Giemsa staining. For histopathologic analyses, femurs were fixed for more than 24 hours in 10% neutral-buffered formalin at room temperature and demineralized in 10% EDTA for 1–2 weeks. The specimens and other soft tissues (spleen and liver) were dehydrated using ethanol and cleared in xylenes. The specimens were then embedded in melted paraffin and allowed to harden. Thin sections (4–5 μm) were cut and floated onto microscope slides. For routine assessment, slides were stained with H&E. For myeloperoxidase staining, the tissue was rehydrated followed by heat-induced epitope retrieval, peroxidase, and serum blocking. Samples were then incubated with myeloperoxidase antibody (R&D Systems) overnight at 4°C followed by staining with the biotinylated second antibody. The slides were visualized under a Keyence BZ-X810 microscope.

### Flow cytometry, cell sorting, and colony assays.

Total white blood cells were obtained after lysis of PB with red cell lysis buffer. Single-cell suspensions from PB and BM were stained with panels of fluorochrome-conjugated antibodies (Supplemental Table 6). Flow cytometric analysis of HSPCs was performed as previously described (53). The analyses were performed using a BD FACS Celesta flow cytometer (BD Biosciences). All data were analyzed by FlowJo 10 software (Tree Star). For Lin^–^cKit^+^ (LK) cell selection, magnetic-activated cell sorting was applied. BM cells were first isolated using the Direct Lineage Cell Depletion kit (Miltenyi Biotec), and then the lineage-negative cells were sorted with cKit (CD117) MicroBeads (Miltenyi Biotec). The purity of selected cells was routinely over 95%. For CFU assays, BM cells were plated in triplicate in methylcellulose medium (Methocult M3134, STEMCELL Technologies) supplemented with 100 ng/mL mouse SCF (mSCF), 10 ng/mL mouse IL-3 (mIL-3), 50 ng/mL mouse TPO (mTPO), 10 ng/mL mouse GM-CSF (mGM-CSF), 4 U/mL human erythropoietin (hEPO), and 50 ng/mL hIL-6. The number of colonies was counted 7 days after seeding. For human cell lines, K562 or Kasumi-1 cells were plated in methylcellulose medium, and the number of colonies was counted 7 days (K562) or 14 days (Kasumi-1) after seeding.

### Transplantation assay.

Competitive repopulation assay was performed by transplantation of a mixture of 1 × 10^6^ four-week-old BoyJ (CD45.1^+^) BM competitor cells and 1 × 10^6^ four-week-old *Mx1Cre^+^* (WT), *Asxl1^Y588X^*Tg, *Mx1Cre^+^* *Kdm6b^fl/+^*, or *Asxl1^Y588X^*Tg *Mx1Cre^+^* *Kdm6b^fl/+^* (CD45.2^+^) BM cells into lethally irradiated (800 cGy) 6- to 8-week-old female BoyJ recipients (CD45.1^+^) by tail vein injection. One month after the transplantation, pI:pC (10 mg/kg) was injected in the recipient mice to induce *Kdm6b* deletion. Flow cytometric analyses were performed to monitor the chimerism by examining the percentages of CD45.1^+^ and CD45.2^+^ cells over a 28-week period after pI:pC injection.

### GSK-J4 in vitro treatment.

BM cells from WT and *Asxl1^Y588X^*Tg mice were plated in methylcellulose medium supplemented with mSCF, mIL-3, mTPO, mGM-CSF, hEPO, and hIL-6 in the presence of either DMSO (vehicle) or various concentrations of GSK-J4 (2.5, 5, 7.5, and 10 μM; Selleck Chemicals). For human leukemia cell lines, 2,000 cells were plated in methylcellulose medium with the addition of 5 μM GSK-J4 or vehicle control. All colonies were scored after 7 days of culture. For CFU assays using human primary cells, the mononuclear cells isolated from PB or BM were seeded into methylcellulose medium supplemented with 100 ng/mL of hSCF, hTPO, hIL-3, hIL-6, hFlt-3L, and 0.75 μM StemRegenin 1 in the presence of 5 μM GSK-J4. The colonies were counted after 14 days of culture at 37°C in a 5% CO_2_ incubator. For cell viability assays, human leukemia cells were plated on 96-well assay plates and treated with GSK-J4 (1, 2, 5, and 10 μM) or vehicle control for 48 and 72 hours, respectively. Cell viability was assessed using CellTiter-Glo 2.0 (Promega) based on the protocol provided by the manufacturer.

### In vivo treatment studies.

2 × 10^6^ spleen cells from *Asxl1^Y588X^*Tg leukemic mice were transplanted into sublethally irradiated (600 cGy) 8-week-old female BoyJ recipient mice (CD45.1^+^). Two weeks after the transplantation, the mice were randomized into 2 groups that received either vehicle or 50 mg/kg GSK-J4 treatment by intraperitoneal injection. The recipient mice were analyzed for hematopoietic phenotypes after 5 weeks of treatment.

For the human leukemia xenograft models, 1 × 10^6^ K562 cells or 1 × 10^4^ THP-1 cells were injected via tail vein into 8-week-old NSG mice following a sublethal irradiation at a dose of 250 cGy. Ten days (THP-1) or 20 days (K562) after the transplantation, GSK-J4 (50 mg/kg) or vehicle was administered into the recipient mice via intraperitoneal injection (5 injections each week). Mouse weight was monitored every day to check toxicity. The survival time of each mouse was recorded, and the moribund mice were analyzed for hematopoietic phenotype.

In addition, two AML PDX cells, #1 (*ASXL1* G646WfsX12, variant allele frequency 39%) and #2 (no known *ASXL1* mutation), were transplanted into sublethally irradiated (300 cGy) 8- to 10-week-old NSG mice by intravenous injection. For both models, 10 days after the transplantation, the mice were randomized into 2 groups that received either vehicle or 50 mg/kg GSK-J4 treatment by intraperitoneal injection. Mouse weight was monitored daily to check toxicity. Mice were sacrificed when moribund, and human CD45 chimerism and spleen weights were measured to assess disease burden.

### Quantitative PCR analysis.

Total RNA was extracted with TRIzol reagent (Invitrogen), and cDNA was synthesized using the QuantiTect reverse transcription kit (Qiagen) according to the manufacturer’s instructions. qPCR was performed in triplicate using an Applied Biosystems QuantStudio 3 system with the Fast SYBR Green master mix (Applied Biosystems). The expression of gene of interest was calculated using the 2^–ΔΔCt^ method by normalization to the housekeeping gene (*β-actin*). All qPCR primers used are listed in Supplemental Table 4.

### Western blot assay.

The whole-cell lysates were prepared using the RIPA buffer (MilliporeSigma) and then resolved on NuPAGE 4%–12% Bis-Tris Gels (Invitrogen). Immunoblotting was performed with the following antibodies (Supplemental Table 6): rabbit monoclonal anti-EZH2 (1:1,000), rabbit monoclonal anti-SUZ12 (1:1,000), rabbit monoclonal anti-EED (1:1,000), mouse monoclonal anti–β-actin (1:2,000), rabbit monoclonal anti-KDM6A (1:1,000), rabbit polyclonal anti-KDM6B (1:1,000), rabbit polyclonal anti-H3K27me2 (1:1,000), rabbit polyclonal anti-H3K27me3 (1:1,000), rabbit polyclonal anti-H3 (1:4,000), and mouse monoclonal anti-FLAG (1:1,000). After incubation with anti-rabbit IgG or anti-mouse IgG antibodies conjugated with horseradish peroxidase (GE Healthcare), signals were detected using Prometheus ProSignal ECL reagents (Genesee Scientific). Images were taken on a ChemiDoc MP Imaging System (Bio-Rad Laboratories).

### RNA-Seq and data analysis.

BM LK cells were purified from 12-month-old *Asxl1^Y588X^*Tg preleukemic mice and age-matched WT, *Kdm6b^Δ/+^*, and *Asxl1^Y588X^*Tg *Kdm6b^Δ/+^* mice. Total RNA from individual mice was isolated with RNeasy Plus Mini Kit (Qiagen). Approximately 500 ng RNA was used for library preparation, following the KAPA Stranded RNA-Seq Kit with RiboErase (HMR) sample preparation guide (KAPA Biosystems). The libraries were subsequently sequenced with 50 bp single-read sequencing run using Illumina HiSeq 3000 platform.

The raw data were trimmed by Trimmomatic (v0.38) (54), and approximately 20 million to 30 million reads per sample were aligned to the mouse genome (mm10) using STAR (v2.7.0e) (55). The raw read counts of each gene were calculated by HTSeq (v0.11.2) (56) and converted to transcripts per million format. Then, the count matrix was used to identify differentially expressed genes by pairwise comparison using DESeq2 (57) with a cutoff of false discovery rate (FDR) < 0.05 and |fold change| > 1.5. Transcripts per million was used for GSEA (58).

### ChIP sequencing and data analysis.

Chromatin immunoprecipitation (ChIP) assays were performed using LK cells from 10- to 12-month-old mice of the 4 different genotypes, as previously reported (53). Genomic DNA regions of interest were isolated using antibody against H3K27me3 (07-449, MilliporeSigma). The ChIP libraries were prepared using the MicroPlex library preparation kit v3 (Diagenode) and sequenced with a read length of 75 bp on an Illumina NextSeq 500 system.

Raw data of each sample were filtered and trimmed by TrimGalore (v0.5.0, https://github.com/FelixKrueger/TrimGalore). Clean reads were aligned to the mouse reference genome (mm10) using Bowtie 2 (v2.3.4.3) (59, 60). The raw SAM files generated by Bowtie 2 were sorted using SAMtools (v1.9) (61). PCR duplications were removed by Sambamba (v0.6.8) (62). Peak calling was performed with MACS2 (v2.1.2) (63) with options: -g mm -B -q 0.05; and the total numbers of H3K27me3 peaks were identified (WT: #1, 61295; #2, 55267; *Kdm6b^Δ/+^*: #1, 45288; #2, 50968; *Asxl1^Y588X^*Tg: #1, 18088; #2, 37427; *Asxl1^Y588X^*Tg *Kdm6b^Δ/+^*: #1, 50870; #2, 38826). The BAM file was normalized using deepTools (v3.1.3) (64) to generate a BigWig file for visualization with options: --normalizeUsing CPM --binSize 50. Differential peaks were detected by DiffBind (65) using the embedded method edgeR with a threshold of FDR ≤ 0.05. ChIPpeakAnno (66) and ChIPseeker (67) were used to annotate the total or differential peaks. All figures were visualized in R and GraphPad Prism software.

### Statistics.

Differences between experimental groups were determined by the log-rank test, Mann-Whitney *U* test, unpaired or paired 2-tailed Student’s *t* test, or 1-way ANOVA followed by an appropriate post hoc correction. *P* values less than 0.05 were considered significant. Statistical analyses were conducted and analyzed using GraphPad Prism 9.0 (GraphPad Software). The statistical methods used for comparisons are indicated in the relevant figure legends.

### Study approval.

The institutional review board of the University of Texas Health Science Center at San Antonio (protocol 20220335NHR), in accordance with the Declaration of Helsinki, approved the use of deidentified leftover myeloid malignancy patient specimens (i.e., PB or BM aspirates) and associated clinical data. The samples were not collected specifically for the current experiments through an interaction or intervention with living individuals. All animal experiments in this investigation were conducted in accordance with the National Institutes of Health’s guidelines on animal care and use. Experiment protocols were approved by the Institutional Animal Care and Use Committee of the University of Texas Health Science Center at San Antonio. All animals received humane care in compliance with the National Institutes of Health *Guide for the Care and Use of Laboratory Animals* (National Academies Press, 2011).

### Data availability.

The RNA-Seq and ChIP-Seq data in this study were deposited in the NCBI’s Gene Expression Omnibus (GSE208249). Values for all data points in graphs are reported in the Supporting Data Values file.

## Author contributions

PZ, GG, and FCY conceived the project and designed the study. GG, PZ, SC, HY, YG, IPR, AN, CRD, and HG performed the experiments. PS, PZ, and GG performed the RNA-Seq and ChIP-Seq analyses. GG, PZ, PS, JAP, EAM, YL, SDN, RM, OAW, MX, and FCY discussed and analyzed the data. PZ, GG, and FCY wrote the manuscript with help from other authors. FCY initiated and supervised the project. All authors read and approved the final manuscript.

## Supplementary Material

Supplemental data

Supporting data values

## Version 1 11/02/2023

In-Press Preview

## Version 2 01/02/2024

Electronic publication

## References

[1] Gelsi-Boyer V (2009). Mutations of polycomb-associated gene ASXL1 in myelodysplastic syndromes and chronic myelomonocytic leukaemia. Br J Haematol.

[2] Gelsi-Boyer V (2010). ASXL1 mutation is associated with poor prognosis and acute transformation in chronic myelomonocytic leukaemia. Br J Haematol.

[3] Bejar R (2011). Clinical effect of point mutations in myelodysplastic syndromes. N Engl J Med.

[4] Thol F (2011). Prognostic significance of ASXL1 mutations in patients with myelodysplastic syndromes. J Clin Oncol.

[5] Carbuccia N (2009). Mutations of ASXL1 gene in myeloproliferative neoplasms. Leukemia.

[6] Brecqueville M (2012). Mutation analysis of ASXL1, CBL, DNMT3A, IDH1, IDH2, JAK2, MPL, NF1, SF3B1, SUZ12, and TET2 in myeloproliferative neoplasms. Genes Chromosomes Cancer.

[7] Caye A (2015). Juvenile myelomonocytic leukemia displays mutations in components of the RAS pathway and the PRC2 network. Nat Genet.

[8] Stieglitz E (2015). The genomic landscape of juvenile myelomonocytic leukemia. Nat Genet.

[9] Chou WC (2010). Distinct clinical and biological features of de novo acute myeloid leukemia with additional sex comb-like 1 (ASXL1) mutations. Blood.

[10] Patel JP (2012). Prognostic relevance of integrated genetic profiling in acute myeloid leukemia. N Engl J Med.

[11] Gelsi-Boyer V (2012). Mutations in ASXL1 are associated with poor prognosis across the spectrum of malignant myeloid diseases. J Hematol Oncol.

[12] Inoue D (2016). Truncation mutants of ASXL1 observed in myeloid malignancies are expressed at detectable protein levels. Exp Hematol.

[13] Genovese G (2014). Clonal hematopoiesis and blood-cancer risk inferred from blood DNA sequence. N Engl J Med.

[14] Jaiswal S (2014). Age-related clonal hematopoiesis associated with adverse outcomes. N Engl J Med.

[15] Xie M (2014). Age-related mutations associated with clonal hematopoietic expansion and malignancies. Nat Med.

[16] Abdel-Wahab O (2013). Deletion of Asxl1 results in myelodysplasia and severe developmental defects in vivo. J Exp Med.

[17] Wang J (2014). Loss of Asxl1 leads to myelodysplastic syndrome-like disease in mice. Blood.

[18] Inoue D (2013). Myelodysplastic syndromes are induced by histone methylation–altering ASXL1 mutations. J Clin Invest.

[19] Yang H (2018). Gain of function of ASXL1 truncating protein in the pathogenesis of myeloid malignancies. Blood.

[20] Nagase R (2018). Expression of mutant Asxl1 perturbs hematopoiesis and promotes susceptibility to leukemic transformation. J Exp Med.

[21] Asada S (2018). Mutant ASXL1 cooperates with BAP1 to promote myeloid leukaemogenesis. Nat Commun.

[22] Abdel-Wahab O (2012). ASXL1 mutations promote myeloid transformation through loss of PRC2-mediated gene repression. Cancer Cell.

[23] Scheuermann JC (2010). Histone H2A deubiquitinase activity of the Polycomb repressive complex PR-DUB. Nature.

[24] Li Z (2017). ASXL1 interacts with the cohesin complex to maintain chromatid separation and gene expression for normal hematopoiesis. Sci Adv.

[25] Shi H (2016). ASXL1 plays an important role in erythropoiesis. Sci Rep.

[26] Hong S (2007). Identification of JmjC domain-containing UTX and JMJD3 as histone H3 lysine 27 demethylases. Proc Natl Acad Sci U S A.

[27] Swigut T, Wysocka J (2007). H3K27 demethylases, at long last. Cell.

[28] De Santa F (2007). The histone H3 lysine-27 demethylase Jmjd3 links inflammation to inhibition of polycomb-mediated gene silencing. Cell.

[29] Agger K (2007). UTX and JMJD3 are histone H3K27 demethylases involved in HOX gene regulation and development. Nature.

[30] Lan F (2007). A histone H3 lysine 27 demethylase regulates animal posterior development. Nature.

[31] Agger K (2009). The H3K27me3 demethylase JMJD3 contributes to the activation of the INK4A-ARF locus in response to oncogene- and stress-induced senescence. Genes Dev.

[32] Sen GL (2008). Control of differentiation in a self-renewing mammalian tissue by the histone demethylase JMJD3. Genes Dev.

[33] Mallaney C (2019). Kdm6b regulates context-dependent hematopoietic stem cell self-renewal and leukemogenesis. Leukemia.

[34] Wei Y (2013). Global H3K4me3 genome mapping reveals alterations of innate immunity signaling and overexpression of JMJD3 in human myelodysplastic syndrome CD34+ cells. Leukemia.

[35] Li Y (2018). Therapeutic potential of GSK-J4, a histone demethylase KDM6B/JMJD3 inhibitor, for acute myeloid leukemia. J Cancer Res Clin Oncol.

[36] Ohguchi H (2017). KDM6B modulates MAPK pathway mediating multiple myeloma cell growth and survival. Leukemia.

[37] Anderton JA (2011). The H3K27me3 demethylase, KDM6B, is induced by Epstein-Barr virus and over-expressed in Hodgkin’s Lymphoma. Oncogene.

[38] Ntziachristos P (2014). Contrasting roles of histone 3 lysine 27 demethylases in acute lymphoblastic leukaemia. Nature.

[39] Mathur R (2017). Inhibition of demethylase KDM6B sensitizes diffuse large B-cell lymphoma to chemotherapeutic drugs. Haematologica.

[40] Black JC (2012). Histone lysine methylation dynamics: establishment, regulation, and biological impact. Mol Cell.

[41] Mosammaparast N, Shi Y (2010). Reversal of histone methylation: biochemical and molecular mechanisms of histone demethylases. Annu Rev Biochem.

[42] Bottomly D (2022). Integrative analysis of drug response and clinical outcome in acute myeloid leukemia. Cancer Cell.

[43] Manna S (2015). Histone H3 Lysine 27 demethylases Jmjd3 and Utx are required for T-cell differentiation. Nat Commun.

[44] Kruidenier L (2012). A selective jumonji H3K27 demethylase inhibitor modulates the proinflammatory macrophage response. Nature.

[45] Shultz LD (2005). Human lymphoid and myeloid cell development in NOD/LtSz-scid IL2R gamma null mice engrafted with mobilized human hemopoietic stem cells. J Immunol.

[46] Menendez-Gonzalez JB (2019). Gata2 as a crucial regulator of stem cells in adult hematopoiesis and acute myeloid leukemia. Stem Cell Reports.

[47] Argiropoulos B (2007). Unraveling the crucial roles of Meis1 in leukemogenesis and normal hematopoiesis. Genes Dev.

[48] Balasubramani A (2015). Cancer-associated ASXL1 mutations may act as gain-of-function mutations of the ASXL1-BAP1 complex. Nat Commun.

[49] Sera Y (2021). UTX maintains the functional integrity of the murine hematopoietic system by globally regulating aging-associated genes. Blood.

[50] Wei Y (2018). KDM6B overexpression activates innate immune signaling and impairs hematopoiesis in mice. Blood Adv.

[51] Youmans DT (2018). Live-cell imaging reveals the dynamics of PRC2 and recruitment to chromatin by SUZ12-associated subunits. Genes Dev.

[52] Heinemann B (2014). Inhibition of demethylases by GSK-J1/J4. Nature.

[53] Zhang P (2018). Chromatin regulator Asxl1 loss and Nf1 haploinsufficiency cooperate to accelerate myeloid malignancy. J Clin Invest.

[54] Bolger AM (2014). Trimmomatic: a flexible trimmer for Illumina sequence data. Bioinformatics.

[55] Dobin A (2013). STAR: ultrafast universal RNA-seq aligner. Bioinformatics.

[56] Anders S (2015). HTSeq—a Python framework to work with high-throughput sequencing data. Bioinformatics.

[57] Love MI (2014). Moderated estimation of fold change and dispersion for RNA-seq data with DESeq2. Genome Biol.

[58] Subramanian A (2005). Gene set enrichment analysis: a knowledge-based approach for interpreting genome-wide expression profiles. Proc Natl Acad Sci U S A.

[59] Langmead B, Salzberg SL (2012). Fast gapped-read alignment with Bowtie 2. Nat Methods.

[60] Langmead B (2019). Scaling read aligners to hundreds of threads on general-purpose processors. Bioinformatics.

[61] Li H (2009). The sequence alignment/map format and SAMtools. Bioinformatics.

[62] Tarasov A (2015). Sambamba: fast processing of NGS alignment formats. Bioinformatics.

[63] Feng J (2012). Identifying ChIP-seq enrichment using MACS. Nat Protoc.

[64] Ramirez F (2014). deepTools: a flexible platform for exploring deep-sequencing data. Nucleic Acids Res.

[65] Ross-Innes CS (2012). Differential oestrogen receptor binding is associated with clinical outcome in breast cancer. Nature.

[66] Zhu LJ (2010). ChIPpeakAnno: a Bioconductor package to annotate ChIP-seq and ChIP-chip data. BMC Bioinformatics.

[67] Yu G (2015). ChIPseeker: an R/Bioconductor package for ChIP peak annotation, comparison and visualization. Bioinformatics.

