# Supplementary material for: Targeting lysine demethylase 6B ameliorates ASXL1 truncation–mediated myeloid malignancies in preclinical models
Source: J Clin Invest. 2024 Jan 2;134(1):e163964. doi: 10.1172/JCI163964 (PMC10760961; doi:10.1172/JCI163964)
Supplement: Supplemental data [file jci-134-163964-s174.pdf]

Supplemental Figure 1

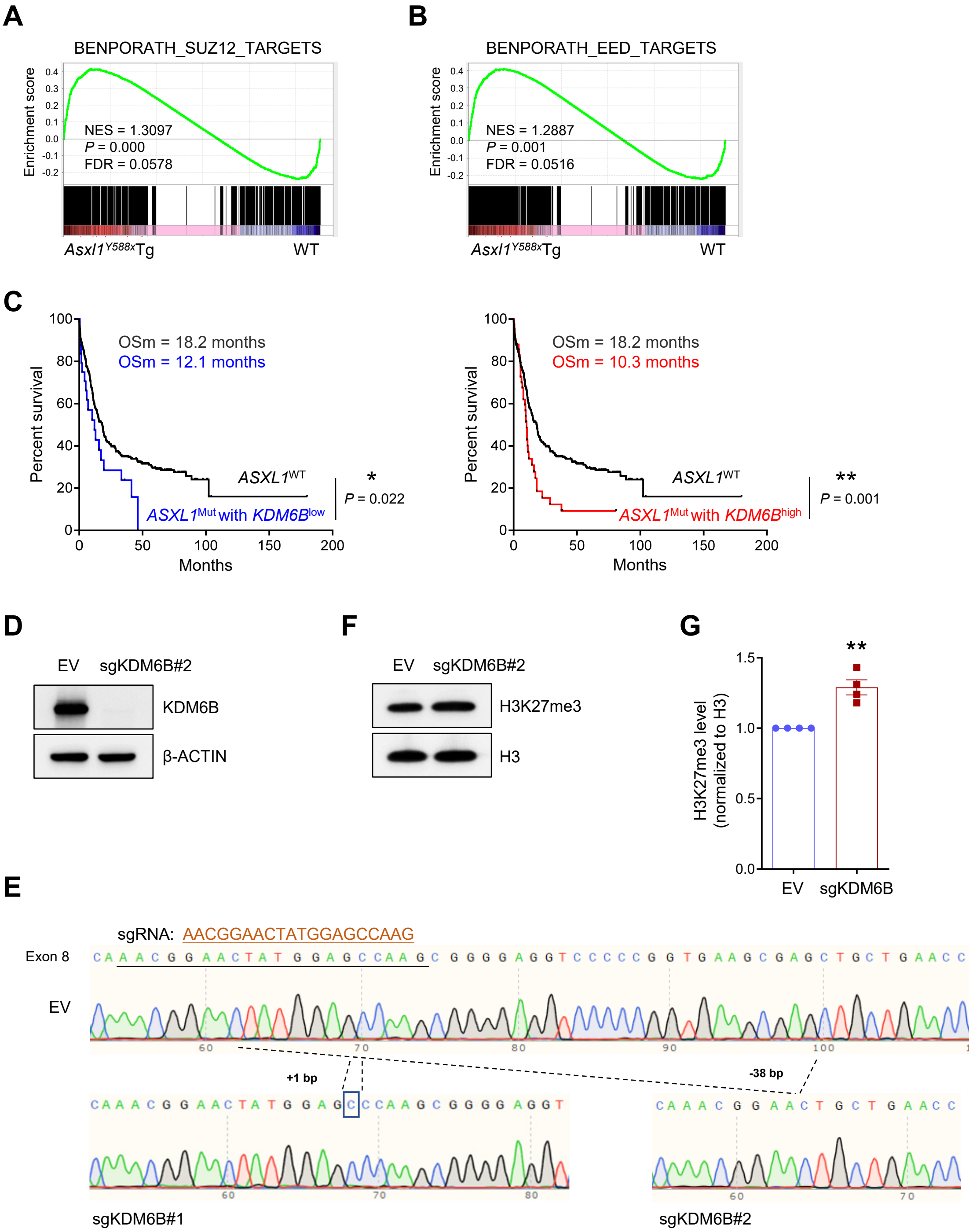

Supplemental Figure 1. KDM6B is up-regulated in *Asxl1* mutant cells. (A and B) Gene set enrichment analysis (GSEA) shows that the targets of SUZ12 (A) and EED (B) are up-regulated in $Asxl1^{Y588X}$Tg LK cells. The normalized enrichment score (NES), *P*-value, and FDR are shown. (C) Kaplan-Meier survival analyses of AML patients with *ASXL1* mutations based on their *KDM6B* expression level. The median level of *KDM6B* in $ASXL1^{\mathrm{WT}}$ was used to determine the expression level of *KDM6B* in $ASXL1^{\mathrm{Mut}}$. OSm, medium overall survival. $ASXL1^{\mathrm{WT}}$, n = 583; $ASXL1^{\mathrm{Mut}}$ with $KDM6B^{\mathrm{low}}$, n = 24; $ASXL1^{\mathrm{Mut}}$ with $KDM6B^{\mathrm{high}}$, n = 42. (D and F) Western blot analysis of KDM6B (D) and H3K27me3 (F) levels in K562 cells after expressing empty vector (EV) or sgRNA targeting KDM6B (sgKDM6B). (E) Sanger sequencing of *KDM6B* locus in K562 cells after nucleofection of EV and sgKDM6B. (G) Four independent Western blots were quantified by densitometry using ImageJ software. Data represent the mean ± SEM. **P* < 0.05 and ***P* < 0.01, by Log-rank (Mantel-Cox) test (C) or unpaired Student's *t* test (G).

Supplemental Figure 2

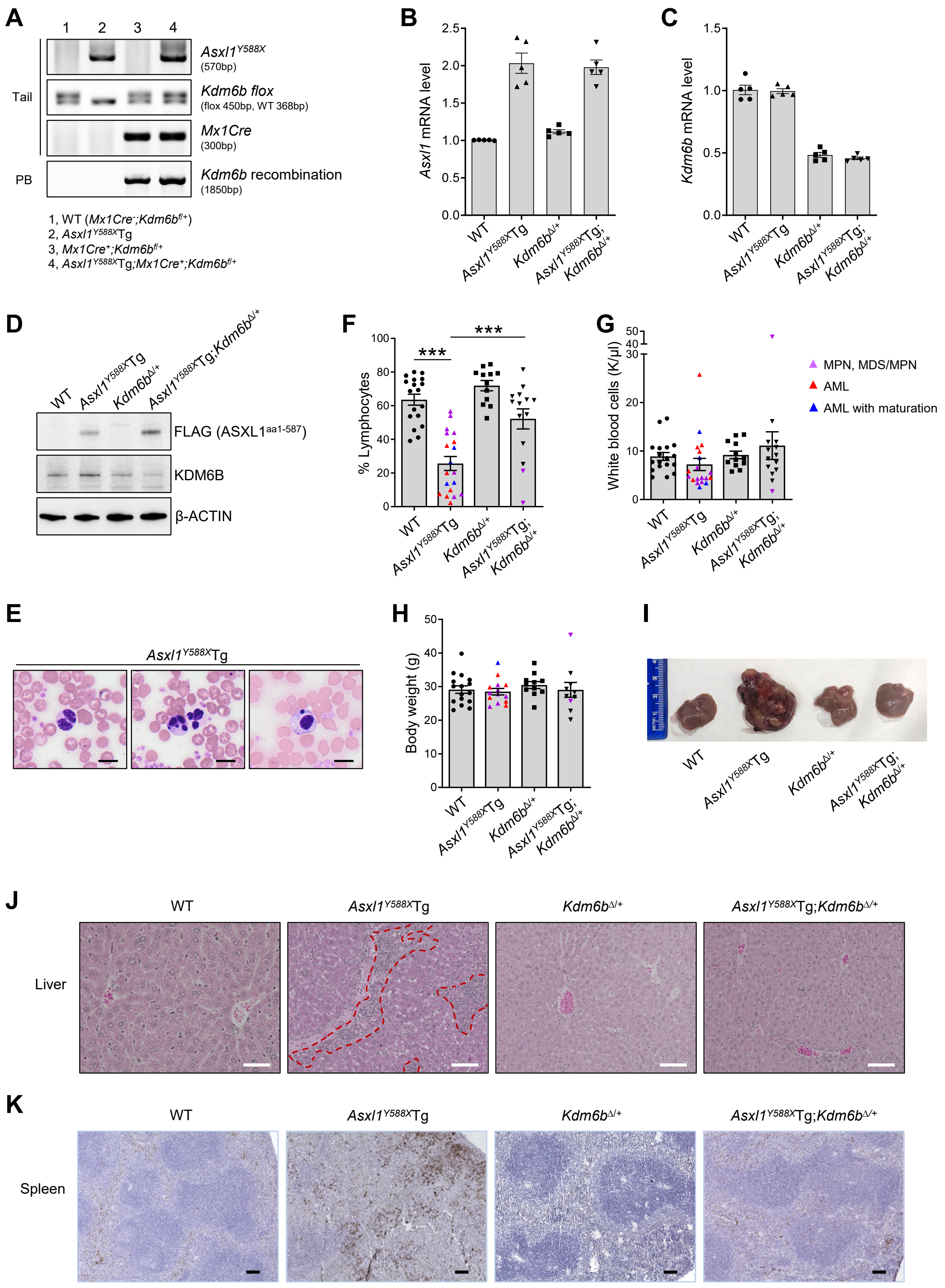

Supplemental Figure 2. Heterozygous deletion of *Kdm6b* blocks the development of ASXL1^{Y588X}-mediated myeloid malignancies. (A) Genotyping results of WT, *Asxl1*^{Y588X}Tg, *Mx1Cre*$^{+}$;*Kdm6b*$^{flox/+}$ (*Kdm6b*$^{\Delta/+}$), and *Asxl1*^{Y588X}Tg;*Mx1Cre*$^{+}$;*Kdm6b*$^{flox/+}$ (*Asxl1*^{Y588X}Tg;*Kdm6b*$^{\Delta/+}$) mice. (B and C) qPCR showing the mRNA levels of *Asxl1* (both endogenous and transgenic mutant, B) and *Kdm6b* (C) in BM cells from WT, *Asxl1*^{Y588X}Tg, *Kdm6b*$^{\Delta/+}$, and *Asxl1*^{Y588X}Tg;*Kdm6b*$^{\Delta/+}$ mice (n = 5 per genotype). (D) Western blots showing ASXL1$^{aa1-587}$ expression and KDM6B protein levels in the BM cells from mice of each genotype. β-ACTIN was used as a loading control. (E) May-Giemsa–stained PB smears demonstrating dysplastic erythroid cells and neutrophils from representative *Asxl1*^{Y588X}Tg mice. Scale bar, 10 μm. (F and G) Percent of lymphocytes (F) and white blood cells count (G) in WT (n = 18), *Asxl1*^{Y588X}Tg (n = 19), *Kdm6b*$^{\Delta/+}$ (n = 12), and *Asxl1*^{Y588X}Tg;*Kdm6b*$^{\Delta/+}$ (n = 14) mice. (H) Body weight from WT (n = 16), *Asxl1*^{Y588X}Tg (n = 14), *Kdm6b*$^{\Delta/+}$ (n = 11), and *Asxl1*^{Y588X}Tg;*Kdm6b*$^{\Delta/+}$ (n = 10) mice are shown. (I) The gross appearance of liver from representative mice of each genotype. (J) Representative H&E staining of liver sections is shown. Scale bar, 100 μm. (K) Representative MPO staining of spleen sections is shown. Scale bar, 100 μm. Data represent the mean ± SEM. ****P* < 0.001, by 1-way ANOVA with Tukey's multiple comparisons test.

Supplemental Figure 3

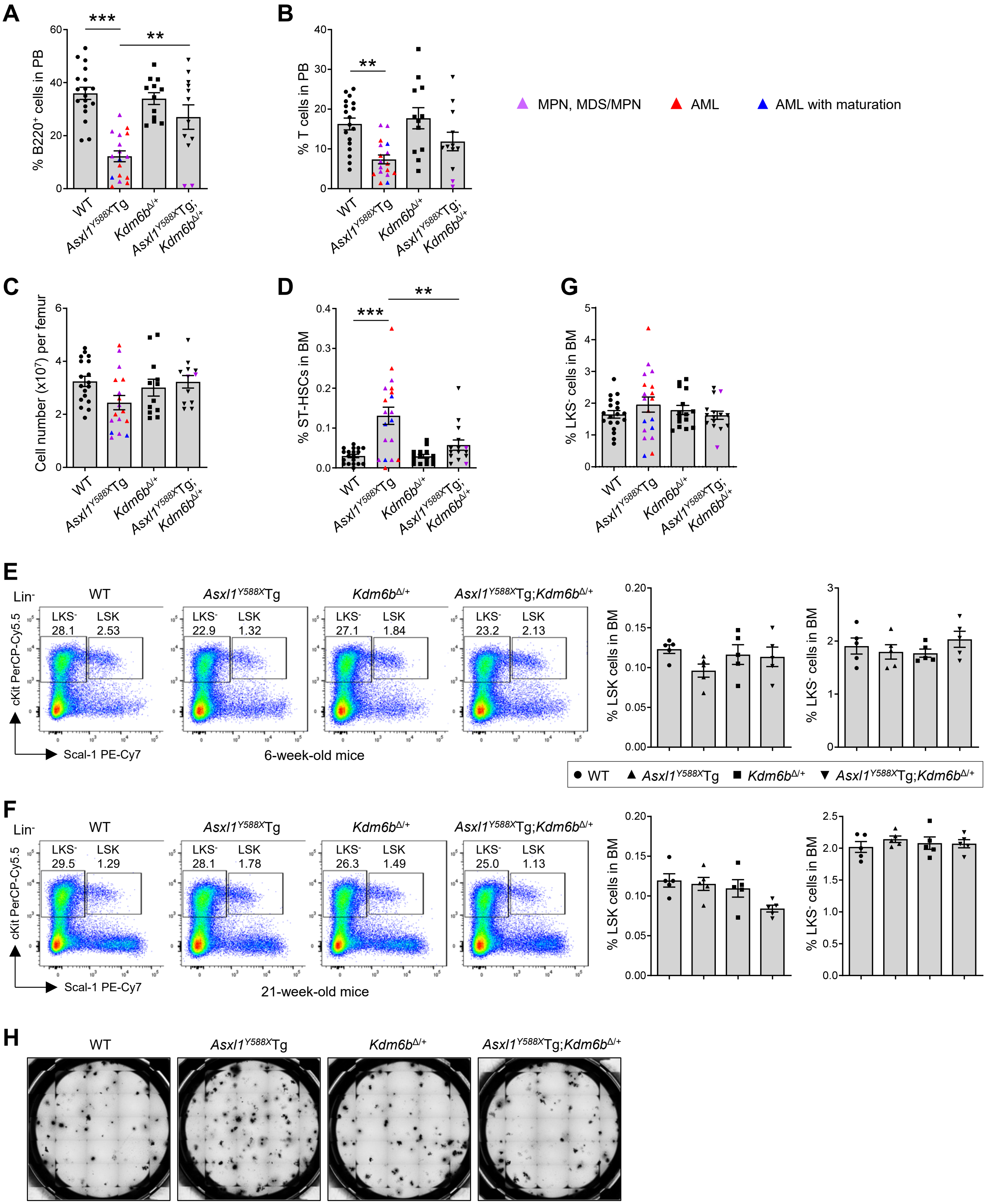

Supplemental Figure 3 (cont'd)

I

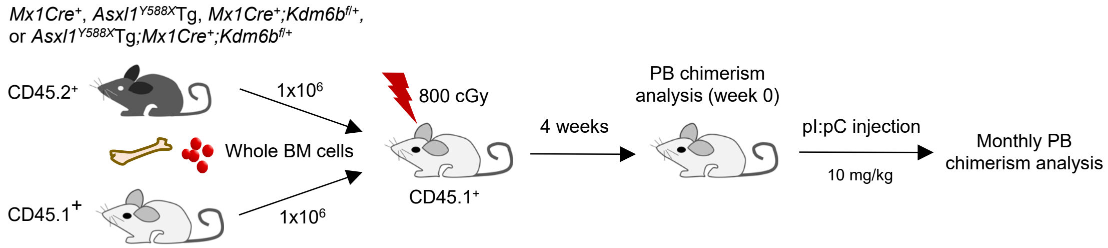

J

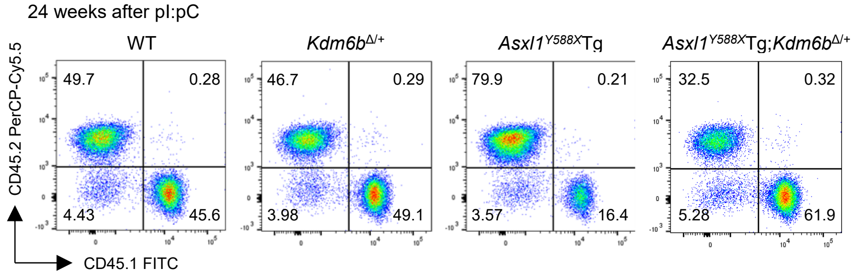

Supplemental Figure 3. Genetic reduction of *Kdm6b* restores ASXL1^{Y588X}-mediated HSC phenotypes and myeloid differentiation. (A and B) Frequencies of B cells (B220$^+$, A) and T cells (CD4$^+$, CD8$^+$, and CD4$^+$/CD8$^+$, B) in PB from WT (n = 18), *Asxl1*^{Y588X}Tg (n = 17), *Kdm6b*$^{\Delta/+}$ (n = 12), and *Asxl1*^{Y588X}Tg;*Kdm6b*$^{\Delta/+}$ (n = 12) mice are shown. (C) BM cellularity of WT (n = 18), *Asxl1*^{Y588X}Tg (n = 17), *Kdm6b*$^{\Delta/+}$ (n = 12), and *Asxl1*^{Y588X}Tg;*Kdm6b*$^{\Delta/+}$ (n = 12) mice. (D and G) Quantification of the percentage of ST-HSCs (d) and LKS$^-$ cells (G) in BM from WT (n = 19), *Asxl1*^{Y588X}Tg (n = 19), *Kdm6b*$^{\Delta/+}$ (n = 15), and *Asxl1*^{Y588X}Tg;*Kdm6b*$^{\Delta/+}$ (n = 15) mice. (E and F) Flow cytometric analysis of HSPCs in BM cells from each genotype at 6 weeks (E) and 21 weeks (F) of age (n = 5 mice per group). (H) Representative images of colony formation for BM cells from each genotype. The images were taken on the seventh day of the assay. (I) Schematic for competitive repopulation assay. CD45.2$^+$ BM cells from WT, *Asxl1*^{Y588X}Tg, *Mx1Cre*$^+$;*Kdm6b*$^{flox/+}$, or *Asxl1*^{Y588X}Tg;*Mx1Cre*$^+$;*Kdm6b*$^{flox/+}$ mice were mixed with equal numbers of CD45.1$^+$ competitor cells and transplanted into lethally irradiated CD45.1$^+$ recipient mice followed by pI:pC injection (10 mg/kg) 4 weeks afterwards. (J) Representative flow cytometric analysis of PB from recipient animals 24 weeks after pI:pC injection. Data represent the mean ± SEM. $^{**}P < 0.01$ and $^{***}P < 0.001$, by 1-way ANOVA with Tukey's multiple comparisons test.

Supplemental Figure 4

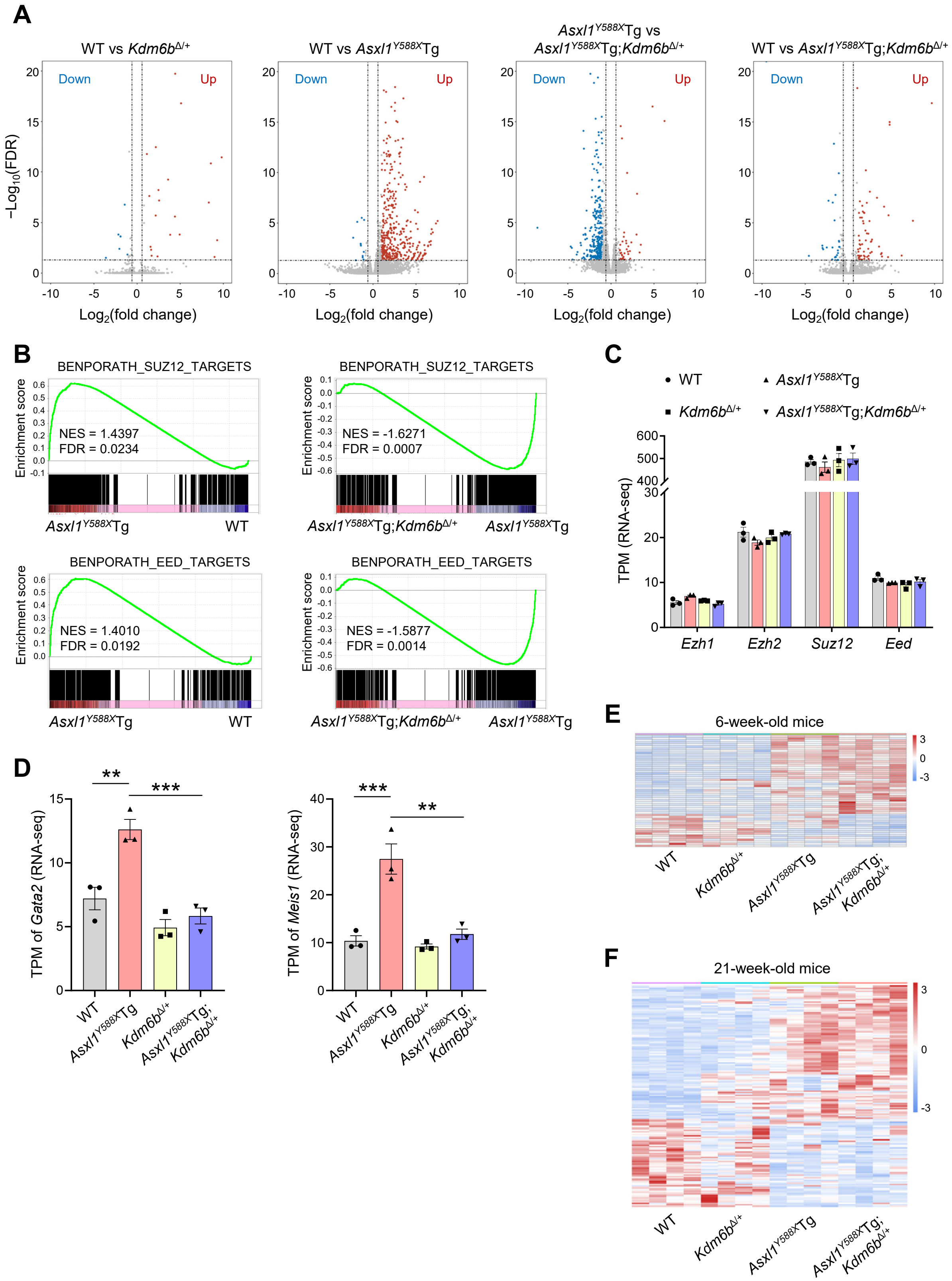

Supplemental Figure 4. Heterozygous deletion of *Kdm6b* decreases ASXL1^{Y588X}-mediated transcription activation in HSPCs. (A) Volcano plot showing the significantly dysregulated genes in Lin$^-$cKit$^+$ (LK) cells from different comparisons (FDR < 0.05 and |fold change| > 1.5). (B) GSEA shows that genes involved in the regulation of SUZ12 and EED targets are up-regulated in *Asxl1*^{Y588X}Tg LK cells, but down-regulated in *Asxl1*^{Y588X}Tg;*Kdm6b*$^{\Delta/+}$ cells. NES, *P* value, and FDR are shown. (C and D) Transcript per million (TPM) values of PRC2 subunits (C), *Gata2* and *Meis1* (D) in RNA-seq are shown (n = 3 per genotype). (E and F) Heatmap displaying gene expression for all genes differentially expressed in LK cells from each mutant genotype relative to WT controls (FDR < 0.05 and |fold change| > 1.5, n = 4 mice per group). Compared with WT cells, the LK cells from 6-week-old *Asxl1*^{Y588X}Tg and *Asxl1*^{Y588X}Tg;*Kdm6b*$^{\Delta/+}$ mice had 21 and 49 dysregulated genes, respectively (E). The numbers of dysregulated genes in 21-week-old *Asxl1*^{Y588X}Tg and *Asxl1*^{Y588X}Tg;*Kdm6b*$^{\Delta/+}$ LK cells were increased to 93 and 116, respectively, compared with WT controls (F). The dysregulated genes were not enriched in HSC function, LSC, and AML pathways. Data represent the mean ± SEM. ***P* < 0.01 and ****P* < 0.001, by 1-way ANOVA with Tukey's multiple comparisons test.

Supplemental Figure 5

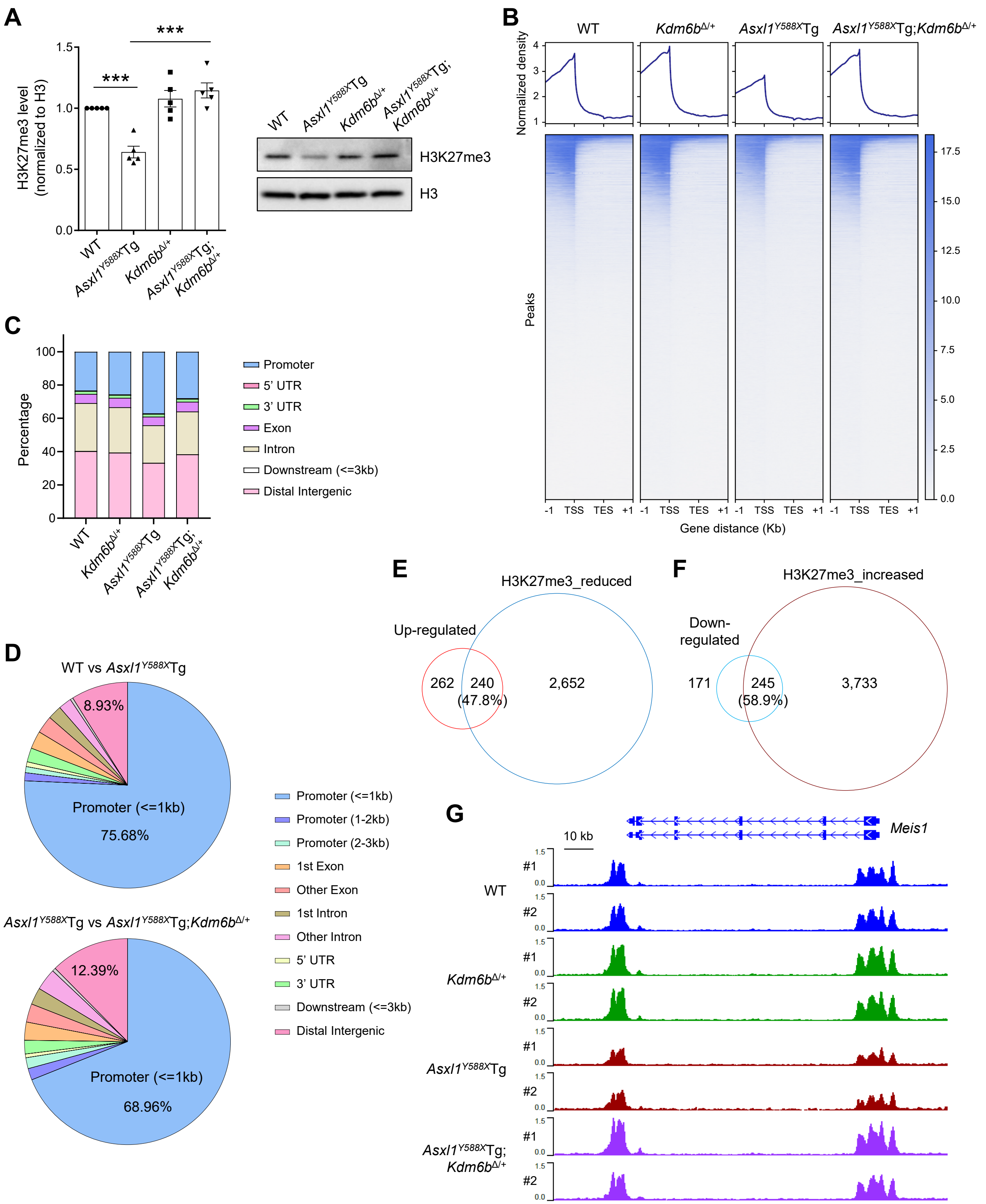

Supplemental Figure 5. Genetic reduction of *Kdm6b* in *Asxl1^{Y588X}*Tg mice normalizes the levels of dysregulated genes by restoring H3K27me3 levels. (A) Western blot analysis and densitometric quantification for H3K27me3 in BM cells from WT, *Asxl1^{Y588X}*Tg, *Kdm6b*$^{\Delta/+}$, and *Asxl1^{Y588X}*Tg;*Kdm6b*$^{\Delta/+}$ mice (n= 5 mice per genotype). Relative H3K27me3 levels were quantified using ImageJ software. (B) Global levels of H3K27me3 at gene body and 1-kb regions surrounding the gene body. The coverages were normalized by the sequencing depth and averaged in two biological replicates. (C) Genome-wide distribution of H3K27me3 peaks in LK cells from WT, *Asxl1^{Y588X}*Tg, *Kdm6b*$^{\Delta/+}$, and *Asxl1^{Y588X}*Tg;*Kdm6b*$^{\Delta/+}$ mice. (D) Genome-wide distribution of differentially enriched H3K27me3 regions in LK cells. Regions that overlap in the two biological replicates are preserved. (E and F) Venn diagram showing the overlaps between dysregulated genes with H3K27me3 changes in LK cells. The number of genes in each section of the diagram is shown. (G) Normalized H3K27me3 signals on the *Meis1* gene loci. Data represent the mean ± SEM. ***P < 0.001, by 1-way ANOVA with Tukey's multiple comparisons test.

Supplemental Figure 6

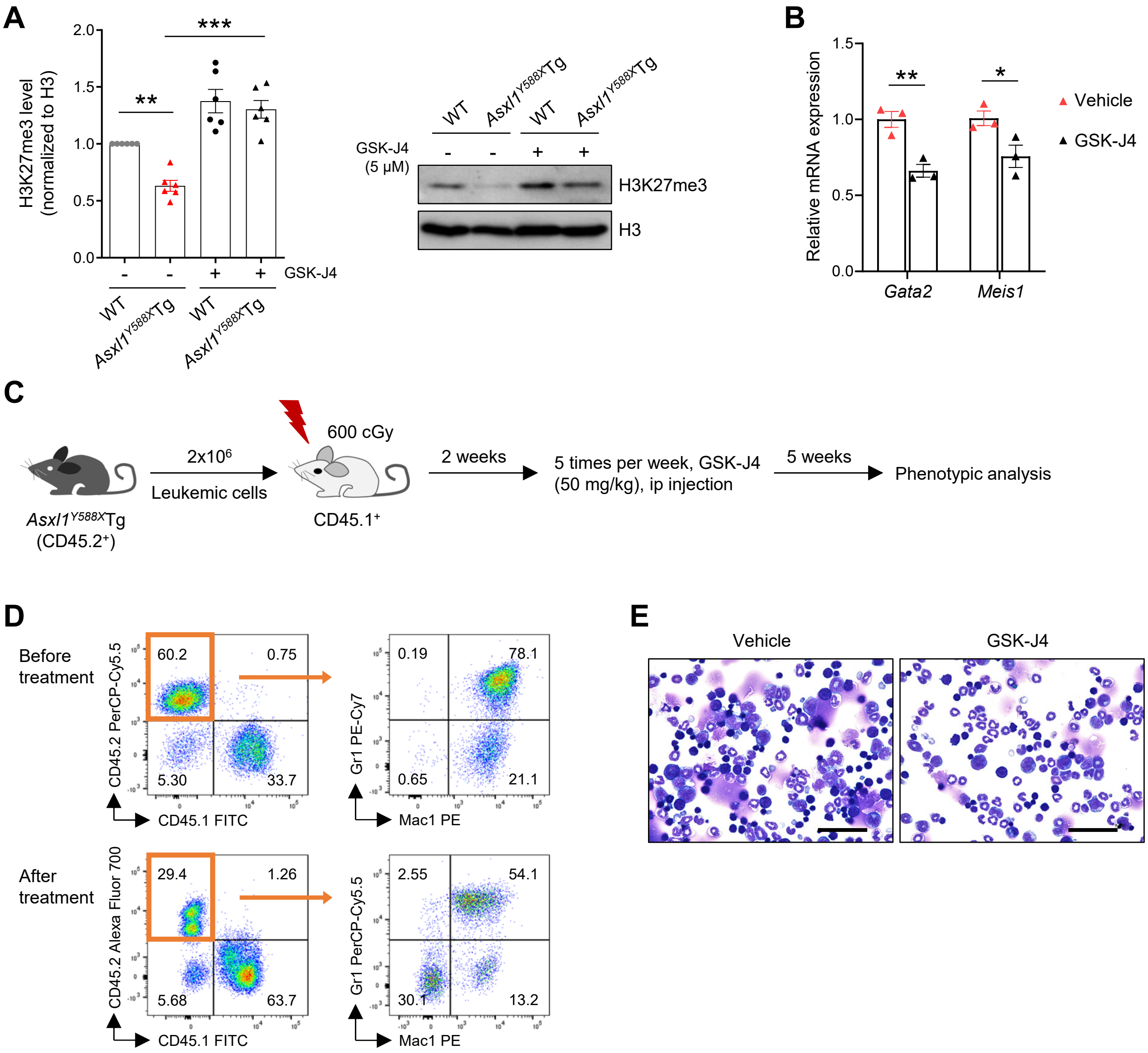

Supplemental Figure 6. *Asxl1^{Y588X}*Tg BM cells are sensitive to KDM6B inhibitor GSK-J4. (A) Western blot and densitometric analysis of H3K27me3 in BM cells with the treatment of 5 μM of GSK-J4 for 24 hours. The quantification was normalized with H3 signal and relative to WT untreated cells. (B) qPCR showing the decreases of *Gata2* and *Meis1* mRNA levels in *Asxl1*^{Y588X}Tg cells treated with GSK-J4 (n = 3). (C) Schema of GSK-J4 treatment *in vivo*. CD45.2^{+} splenic cells from *Asxl1*^{Y588X}Tg leukemic mice were injected into sub-lethally irradiated CD45.1^{+} recipient mice. After 2 weeks of transplantation, the mice were randomized to treated with GSK-J4 or vehicle control. (D) Representative flow cytometric analysis of CD45.2 and Gr1^{+}/Mac1^{+} in BM cells from the recipient mice transplanted with *Asxl1*^{Y588X}Tg cells before and after the treatment of GSK-J4. (E) May-Giemsa–stained BM cytospins prepared from the recipient mice treated with GSK-J4 or vehicle control. Scale bar, 50 μm. Data were derived from 3-4 independent experiments and represent the mean ± SEM. *P < 0.05, **P < 0.01, and ***P < 0.001, by 1-way ANOVA with Tukey's multiple comparisons test (A) or unpaired Student's *t* test (B).

Supplemental Figure 7

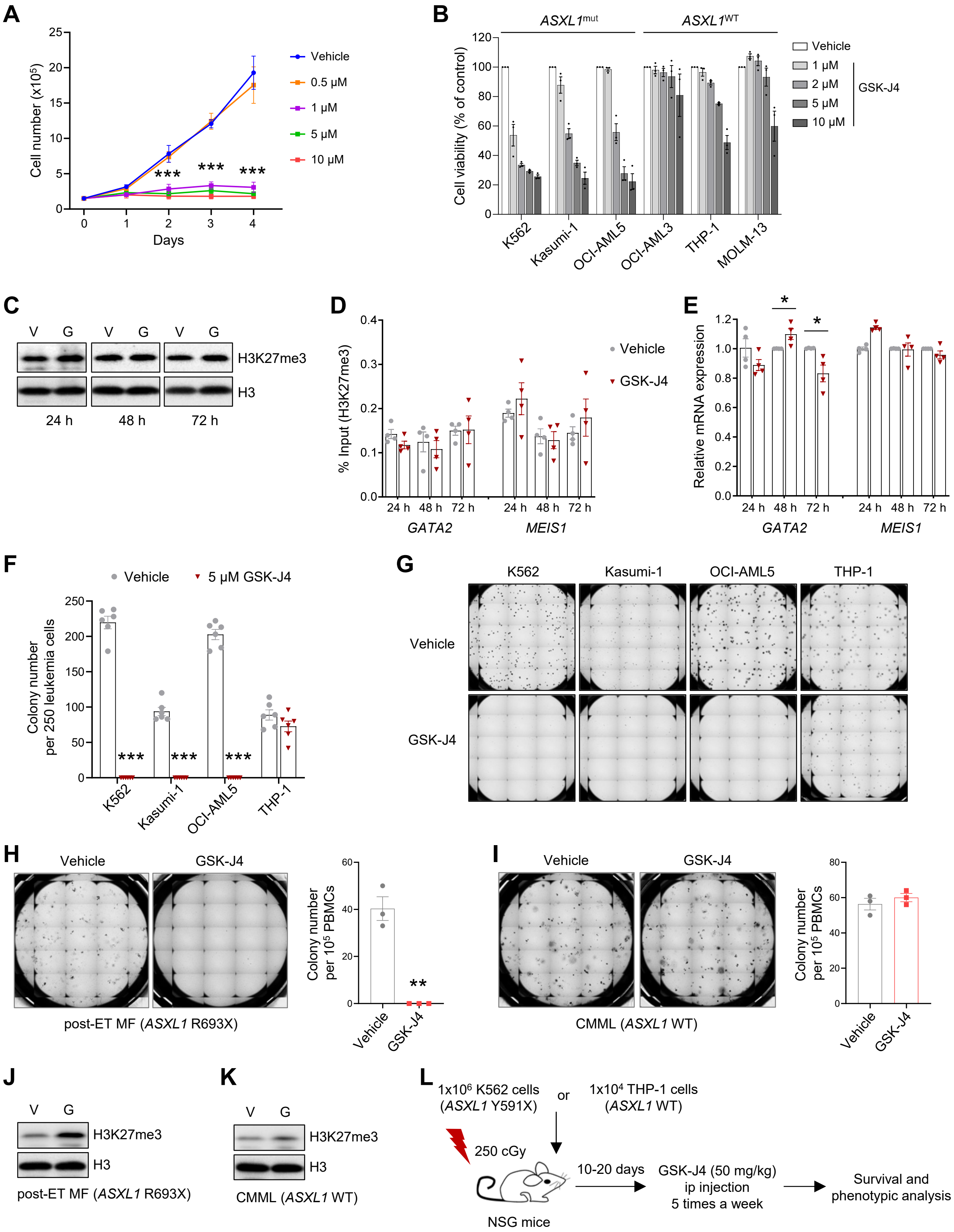

Supplemental Figure 7 (cont'd)

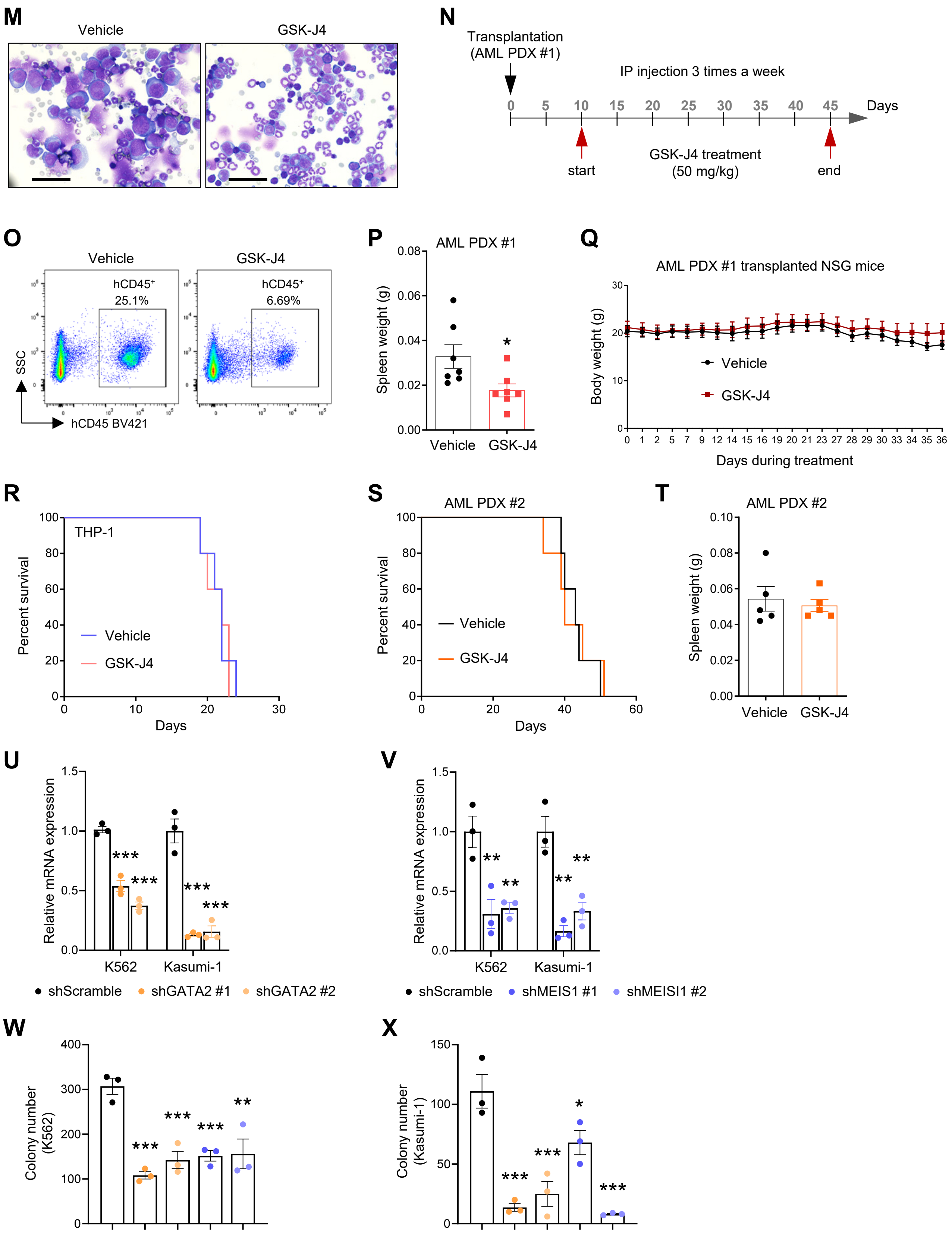

Supplemental Figure 7. Pharmacologic KDM6B inhibition blocks the growth of *ASXL1*-mutation mediated leukemic cells. (A) Proliferation curves of K562 cells treated with GSK-J4 at indicated concentrations. (B) Human leukemia cells were treated with various concentrations of GSK-J4 for 48 hours. The viabilities of the cultured cells were measured using CellTiter-Glo luminescent assay. K562, IC_{50} = 0.406 µM; Kasumi-1, IC_{50} = 2.02 µM; OCI-AML5, IC_{50} = 1.91 µM; OCI-AML3, IC_{50} = 11.77 µM; THP-1, IC_{50} = 8.88 µM; MOLM13, IC_{50} = 9.32 µM. (C) Western blot analysis of H3K27me3 levels in THP-1 cells treated with vehicle control or 5 µM GSK-J4 after 24, 48, and 72 hours, respectively. V, vehicle; G, GSK-J4. (D) ChIP-qPCR showing the levels of H3K27me3 occupancies at the promoter regions of *GATA2* and *MEIS1* in THP-1 cells. (E) The relative mRNA levels of *GATA2* and *MEIS1* in THP-1 cells were analyzed by qPCR. (F and G) Colony-forming assays using human leukemia cell lines in the presence of 5 µM GSK-J4. Representative images of colony formation from each cell line are shown. (H and I) Colony-forming assays using primary patient cells with or without GSK-J4 treatment. Representative images of colony formation for PB cells from a post-ET MF patient (*ASXL1* R693X, H) and a CMML patient (I) are shown. The images were taken on the 14th day of the assay. ET, essential thrombocythemia; MF, myelofibrosis. (J and K) Western blot showing the levels of H3K27me3 in human PB cells from a post-ET MF patient (J) and a CMML patient (K) with the treatment of 5 µM GSK-J4 for 72 hours. (L) Schema of GSK-J4 treatment using human leukemic cell line K562 with *ASXL1* mutation or THP-1 without *ASXL1* mutation. (M) May-Giemsa–stained BM cytospins prepared from the K562-transplanted NSG mice treated with GSK-J4. Scale bar, 50 µm. (N) Schema of GSK-J4 treatment in AML PDX #1 xenografted NSG mice. Black arrow indicating transplantation and red arrows indicating the start/end point of drug treatment. (O) Representative flow cytometric analysis of human CD45 in the BM of NSG mice transplanted with AML PDX #1 after the treatment of GSK-J4 or DMSO. (P) Spleen weights of AML PDX #1 xenografted mice treated with DMSO or GSK-J4 at end point (n = 7 mice per group). (Q) Averaged body weight of AML PDX #1 transplanted NSG mice treated with DMSO or GSK-J4 (n = 7 per group). (R) Kaplan-Meier survival curve for THP-1-transplanted NSG mice treated with DMSO or GSK-J4 (n = 5 per group). (S) Survival curve of NSG mice transplanted with AML PDX #2 and treated with DMSO or 50 mg/kg GSK-J4 (n = 5 per group). (T) Spleen

weights of AML PDX #2 mice shown in S. (U) The mRNA levels of *GATA2* in K562 and Kasumi-1 cells transduced with control shRNA or *GATA2* shRNAs. (V) The mRNA levels of *MEIS1* in K562 and Kasumi-1 cells transduced with control shRNA or *MEIS1* shRNAs. (W and X) Count of colonies formed by K562 (W) and Kasumi-1 (X) cells expressing shRNAs against *GATA2* or *MEIS1*. Data were derived from 3-4 independent experiments and represent the mean ± SEM. **P* < 0.05, ***P* < 0.01, and ****P* < 0.001, by 1-way ANOVA with Tukey's multiple comparisons test (A, U-X) or unpaired Student's *t* test (E, F, H, and P).

Supplemental Table 1. Summary of diseased/moribund *Asxl1*^{Y588X}Tg mice.

Survival (days)	Frequency	BM blasts	Diagnosis and Subclassification
415-800	42.1% (8/19)	<20%	MPN, MDS/MPN
341-751	36.8% (7/19)	>20%	Myeloid leukemia
593-779	21.1% (4/19)	<20%	Myeloid leukemia with maturation

Supplemental Table 2. Summary of diseased *Asxl1*^{Y588X}Tg;*Kdm6b*$^{\Delta/+}$ mice.

Survival (days)	Necropsy and other findings	BM blasts	Diagnosis and Subclassification
590	Hepatosplenomegaly, WBC↑, NE↑, RBC↑, Hgb↑, Plt↓, spleen and liver with myeloid infiltration	<20%	MPN, MDS/MPN
602	WBC↓, NE↑, RBC↓, Hgb↓	<20%	MPN, MDS/MPN

Supplemental Table 3. 109 dysregulated genes involved in HSC functions, LSCs, and AML pathway in *Asxl1*^{Y588X}Tg cells.

#	Gene	log2FC	FDR value	#	Gene	log2FC	FDR value
1	*Clu*	2.70	3.54E-27	56	*Grik5*	1.14	1.60E-03
2	*Col18a1*	3.52	9.66E-26	57	*Hbegf*	1.42	1.68E-03
3	*Mmrn1*	2.21	3.16E-23	58	*Sall2*	1.19	1.76E-03
4	*Hlf*	1.26	7.04E-19	59	*Sh3pxd2b*	0.82	1.82E-03
5	*Dsg2*	3.54	4.43E-18	60	*Muc1*	2.40	1.96E-03
6	*Fgd5*	2.54	7.62E-17	61	*Tnfsf10*	0.81	1.98E-03
7	*Angpt1*	1.47	1.10E-15	62	*Egr3*	1.34	2.06E-03
8	*Mecom*	1.99	3.18E-14	63	*Cdk14*	1.35	2.25E-03
9	*Tgm2*	1.65	4.36E-14	64	*Dlk1*	4.30	2.35E-03
10	*Meis1*	1.26	6.58E-14	65	*Ccdc112*	1.64	2.45E-03
11	*Scarf1*	1.28	1.05E-12	66	*Kcnk5*	1.15	2.46E-03
12	*Slc27a6*	3.05	1.40E-12	67	*Zbtb4*	1.03	2.63E-03
13	*Nrgn*	1.57	1.40E-12	68	*Limch1*	2.80	2.78E-03
14	*Dst*	1.83	1.72E-12	69	*Bex1*	1.89	3.23E-03
15	*Adgrg1*	0.99	1.86E-12	70	*Fut10*	1.11	3.42E-03
16	*Tie1*	1.35	5.92E-12	71	*Kazn*	2.05	3.75E-03
17	*Plxnb2*	1.06	1.31E-11	72	*Adgra3*	0.83	4.25E-03
18	*Fhl1*	2.23	4.86E-11	73	*Gucy1a1*	1.30	4.50E-03
19	*Myct1*	2.04	5.51E-11	74	*Adgrl1*	1.03	4.63E-03
20	*Procr*	2.97	1.41E-09	75	*Chst2*	2.48	5.45E-03
21	*Eya2*	1.95	6.12E-09	76	*Myom1*	1.55	5.74E-03
22	*Ifitm1*	1.34	7.38E-09	77	*Bcl9l*	0.62	6.59E-03
23	*Nbea*	2.06	2.34E-08	78	*Basp1*	0.93	6.99E-03
24	*Obsl1*	2.30	3.48E-08	79	*Prkch*	0.66	8.41E-03
25	*Vdr*	1.38	8.72E-08	80	*Arhgef10*	1.57	8.56E-03
26	*Myo5c*	2.65	1.34E-07	81	*Fzd3*	1.26	9.60E-03
27	*Jag2*	1.90	1.83E-07	82	*Krba1*	0.94	9.82E-03
28	*Jam2*	2.36	3.21E-07	83	*Fstl1*	1.39	9.85E-03
29	*Irf6*	2.26	8.06E-07	84	*Prex2*	1.65	1.07E-02
30	*Ltbp3*	1.18	1.22E-06	85	*Large1*	0.98	1.29E-02
31	*Slc6a15*	1.89	1.55E-06	86	*Abcb1a*	1.96	1.38E-02
32	*Ppp1r9a*	1.23	2.06E-06	87	*Mylk*	0.89	1.57E-02
33	*Prdm16*	1.68	3.55E-06	88	*Pcgf2*	1.17	1.60E-02
34	*H1f0*	0.83	9.09E-06	89	*Arhgef5*	1.09	1.72E-02
35	*Syde1*	2.11	3.37E-05	90	*Fam174b*	0.85	1.76E-02
36	*Sel1l3*	3.43	3.50E-05	91	*Snrpn*	5.79	1.87E-02
37	*Gata2*	0.73	5.24E-05	92	*Cavin3*	2.61	1.92E-02
38	*Ccnd1*	1.03	5.80E-05	93	*Sema3f*	3.26	1.96E-02
39	*Fads1*	0.68	7.67E-05	94	*Eogt*	0.89	2.24E-02
40	*Rorc*	2.41	1.01E-04	95	*Tbxas1*	0.61	2.34E-02
41	*Fgfr1*	1.57	1.58E-04	96	*Mllt3*	0.62	2.73E-02
42	*Ctnnal1*	1.34	1.64E-04	97	*Synpo*	2.60	2.81E-02
43	*Fzd8*	1.69	1.86E-04	98	*Rora*	1.22	2.87E-02
44	*Plekha5*	1.02	1.88E-04	99	*Zfp827*	1.07	2.92E-02
45	*Ocln*	3.87	2.40E-04	100	*Srgap3*	0.77	4.10E-02
46	*Plxnb1*	3.96	4.95E-04	101	*Cacnb2*	0.87	4.11E-02
47	*D630045J12Rik*	1.31	5.72E-04	102	*Zfp467*	1.16	4.37E-02
48	*Adarb1*	2.05	6.13E-04	103	*Nectin1*	0.86	4.50E-02
49	*Crim1*	1.06	6.99E-04	104	*Eid2*	1.35	4.60E-02
50	*Efna1*	5.38	7.57E-04	105	*Ndn*	1.72	4.90E-02
51	*Ifitm3*	0.66	7.66E-04	106	*Cnn3*	-0.85	9.24E-03
52	*Gli3*	5.40	8.44E-04	107	*Mreg*	-0.83	1.61E-02
53	*Hoxb5*	4.30	1.12E-03	108	*Prg2*	-1.09	4.12E-02
54	*Pxdn*	3.21	1.23E-03	109	*Gpc4*	-0.88	4.59E-02
55	*Cntn1*	5.96	1.54E-03	FC, fold change			

Supplemental Table 4. Primers used in this study.

Name	Forward	Reverse
Genotyping PCR (K562)		
KDM6B	CACCCGTGCCATTTTCTCTT	CTGAGAGTGCTGCAGGAGG
Genotyping PCR (mice)		
Asxl1^{Y588X}	ACCCGTCAACGGGACGGAC	CGATCCGGGGGCATATCTGTC
Kdm6b-flox	CAGCGATCCTGACTTGTTCA	GTGCCAAGGCTGGAGGA
Kdm6b-Rec	CGGTCCTGCTACAGTTCTGT	TCTTTGACACGGCCTTGGTA
qPCR		
mAsxl1	TCACACCGAAAAGCCACAG	GGGCATATCTGGTAAGTGGG
mKdm6b	GAGAGGGAGAGTGAGGATGAG	TTGCCTGTGGATGTTACCC
mGata2	ATACCCACCTATCCCTCCTATG	AGCCTTGCTTCTCTGCTTAG
mMeis1	GCAAAGTATGCCAGGGGAGTA	TCCTGTGTTAAGAACCGAGGG
mActb	GGCTGTATTCCCCTCCATCG	CCAGTTGGTAACAATGCCATGT
hGATA2	ACTGACGGAGAGCATGAAGAT	CCGGCACATAGGAGGGGTA
hMEIS1	GGGCATGGATGGAGTAGGC	GGGTACTGATGCGAGTGCAG
hACTB	GCACAGAGCCTCGCCTTT	CGGCGATATCATCATCCAT
ChIP-qPCR		
mGata2	GTCCACAATCCCTAGACTCATG	AGCCCAAATCCAACTGACTC
mMeis1	GTGTGTGGTGTTAGTGCCTG	AGAACCCGGAAGTAGTGGTG
hGATA2	CTCTACCCCCAGCTCCTACC	ACTCGGCCTCTGAGAGTGAA
hMEIS1	GCTGTAAGGACTGTGCCATG	ACAAACACATCGGTTCGCAA

Supplemental Table 5. List of myeloid malignancy patient characteristics.

Primary sample		Cytogenetics
MDS	BMMNC	*ASXL1* c.1934dup, p.Gly646fs; *RUNX1* c.1253dup, p.Met418fs; *U2AF1* c.470A>C, p.Gln157Pro; *CBL* c.1259G>A, p.Arg420Gln; *PHF6* c.71_74del, p.Arg24fs; *TET2* c.3866G>T, p.Cys1289Phe; *TET2* c.3317_3318del, p.Glu1106fs
Post-ET MF	PBMC	*ASXL1* c.2077C>T, p.Arg693*; *JAK2* c.1849G>T, p.Val617Phe
CMML	PBMC	*NPM1* c.860_863dup, p.Trp288fs; *TET2* c.4657C>T, p.Gln1553*; *TET2* c.3322_3328dup, p.Lys1110fs; *NRAS* c.35G>A, p.Gly12Asp; 47,XY,add(7)(q32),+8,add(22)(p13)/46,XY

Supplemental Table 6. Detailed list of reagents used in this study.

Reagent or Resource	Source	Identifier
Antibodies		
Mouse lineage antibody cocktail APC	BD Biosciences	Cat# 558074; RRID: AB_1645213
Rat monoclonal anti-mouse CD117 (cKit) PerCP-Cy5.5	BD Biosciences	Cat# 560557; RRID: AB_1645258
Rat monoclonal anti-mouse Ly-6A/E (Sca1) PE-Cy7	BD Biosciences	Cat# 558162; RRID: AB_647253
Rat monoclonal anti-mouse CD34 FITC	BD Biosciences	Cat# 553733; RRID: AB_395017
Rat monoclonal anti-mouse CD135 BV421	BD Biosciences	Cat# 562898; RRID: AB_2737876
Rat monoclonal anti-mouse CD16/32 APC-Cy7	BioLegend	Cat# 101327; RRID: AB_1967102
Rat monoclonal anti-mouse Ly-6G and Ly-6C (Gr1) PerCP-Cy5.5	BD Biosciences	Cat# 552093; RRID: AB_394334
Rat monoclonal anti-mouse Ly-6G and Ly-6C (Gr1) PE-Cy7	BD Biosciences	Cat# 565033; RRID: AB_2739049
Rat monoclonal anti-mouse CD11b (Mac1) PE	BD Biosciences	Cat# 553311; RRID: AB_394775
Rat monoclonal anti-mouse CD4 PerCP-Cy5.5	BD Biosciences	Cat# 550954; RRID: AB_393977
Rat monoclonal anti-mouse CD4 PE-Cy7	BD Biosciences	Cat# 552775; RRID: AB_394461
Rat monoclonal anti-mouse CD8a PE	BD Biosciences	Cat# 553033; RRID: AB_394571
Rat monoclonal anti-mouse CD45R/B220 APC	BD Biosciences	Cat# 553092; RRID: AB_398531
Mouse monoclonal anti-mouse CD45.2 PerCP-Cy5.5	BD Biosciences	Cat# 552950; RRID: AB_394528
Mouse monoclonal anti-mouse CD45.2 Alexa Fluor 700	Thermo Fisher Scientific	Cat# 56-0454-82; RRID: AB_657752
Mouse monoclonal anti-mouse CD45.1 FITC	BD Biosciences	Cat# 553775; RRID: AB_395043
Mouse monoclonal anti-Human CD45 BV421	BD Horizon	Cat# 563879; RRID: AB_2744402
Mouse monoclonal anti-Myeloperoxidase/MPO	R&D systems	Cat# MAB3174
Rabbit polyclonal anti-H3K27me2	Cell Signaling Technology	Cat# 9728; RRID: AB_1281338
Rabbit polyclonal anti-H3K27me3	MilliporeSigma	Cat# 07-449; RRID: AB_310624
Rabbit polyclonal anti-H3	Abcam	Cat# ab1791; RRID: AB_302613
Rabbit monoclonal anti-EZH2	Cell Signaling Technology	Cat# 5246; RRID: AB_10694683
Rabbit monoclonal anti-SUZ12	Cell Signaling Technology	Cat# 3737; RRID: AB_2196850

Rabbit monoclonal anti-EED	Cell Signaling Technology	Cat# 85322
Rabbit monoclonal anti-KDM6A	Cell Signaling Technology	Cat# 33510; RRID: AB_2721244
Rabbit polyclonal anti-KDM6B	Thermo Fisher Scientific	Cat# PA5-72751; RRID: AB_2718605
Rabbit polyclonal anti-KDM6B	Cell Signaling Technology	Cat# 3457; RRID: AB_1549620
Mouse monoclonal anti-β-Actin	MilliporeSigma	Cat# A2228; RRID: AB_476697
Mouse monoclonal anti-FLAG M2	MilliporeSigma	Cat# F3165; RRID: AB_259529
Mouse IgG, HRP-linked whole Ab (from sheep)	GE Healthcare	Cat# NA931; RRID: AB_772210
Rabbit IgG, HRP-linked $F(ab')_2$ fragment (from donkey)	GE Healthcare	Cat# NA9340; RRID: AB_772191
Bacterial Strains		
One Shot Stbl3 Chemically Competent E. coli	Thermo Fisher Scientific	Cat# C737303
Chemicals, Peptides, and Recombinant Proteins		
Poly(I:C)	InvivoGen	Cat# tlrl-pic-5
GSK-J4	Selleck Chemicals	Cat# S7070
Methylcellulose medium MethoCult M3134	STEMCELL Technologies	Cat# 03134
Recombinant Murine Stem Cell Factor	Peprotech	Cat# 250-03
Recombinant Murine Interleukin 3	Peprotech	Cat# 213-13
Recombinant Murine Thrombopoietin	Peprotech	Cat# 315-14
Recombinant Murine Granulocyte-Macrophage Colony-Stimulating Factor	Peprotech	Cat# 315-03
Recombinant Human Erythropoietin	Peprotech	Cat# 100-64
Recombinant Human Interleukin-6	Peprotech	Cat# 200-06
Recombinant Human SCF	Peprotech	Cat# 300-07
Recombinant Human TPO	Peprotech	Cat# 300-18
Recombinant Human IL-3	Peprotech	Cat# 200-03
Recombinant Human Flt3-Ligand	Peprotech	Cat# 300-19
Recombinant Human GM-CSF	Peprotech	Cat# 300-03
StemRegenin 1	STEMCELL Technologies	Cat# 72342
Mouse CD117 MicroBeads	Miltenyi Biotec	Cat# 130-091-224
Mouse Direct Lineage Cell Depletion Kit	Miltenyi Biotec	Cat# 130-110-470
Lipofectamine 3000 Transfection Reagent	Thermo Fisher Scientific	Cat# L3000075
SF Cell Line 4D-Nucleofector X Kit L	Lonza	Cat#: V4XC-2012
TRIzol Reagent	Thermo Fisher Scientific	Cat# 15596026
RIPA lysis buffer	MilliporeSigma	Cat# 20-188
Prometheus ProSignal Pico ECL Reagent	Genesee Scientific	Cat# 20-300
Prometheus ProSignal Femto ECL Reagent	Genesee Scientific	Cat# 20-302
Critical Commercial Assays		

QIAfilter Plasmid Maxi Kit	Qiagen	Cat# 12263
RNeasy Plus Mini Kit	Qiagen	Cat# 74134
QuantiTect Reverse Transcription Kit	Qiagen	Cat# 205313
Fast SYBR Green Master Mix	Thermo Fisher Scientific	Cat# 4385617
KAPA Stranded RNA-Seq Kit with RiboErase (HMR)	KAPA Biosystems	Cat# KR1151
MicroPlex Library Preparation Kit (v3)	Diagenode	Cat# C05010001
CellTiter-Glo 2.0 Cell Viability Assay	Promega	Cat# G9242
Experimental Models: Cell Lines		
Human: HEK 293TN cell	System Biosciences	Cat# LV900A-1
Human: BM $CD34^+$ cell	Lonza	Cat# 2M-101C
Human: K562 cell	ATCC	Cat# CCL-243
Human: Kasumi-1 cell	ATCC	Cat# CRL-2724
Human: THP-1 cell	ATCC	Cat# TIB-202
Human: MOLM-13 cell	DSMZ	Cat# ACC 554
Human: OCI-AML5 cell	DSMZ	Cat# ACC 247
Human: OCI-AML3 cell	DSMZ	Cat# ACC 582
Experimental Models: Organisms/Strains		
Mouse: B6.Cg-Tg(Mx1-cre)1Cgn/J (Mx1-Cre)	The Jackson Laboratory	JAX: 003556; RRID: IMSR_JAX:003556
Mouse: B6.SJL-$Ptprc^a$ $Pepc^b$/BoyJ (BoyJ)	The Jackson Laboratory	JAX: 002014; RRID: IMSR_JAX:002014
Mouse: B6.Cg-$Kdm6b^{tm1.1Rbo}$/J	The Jackson Laboratory	JAX: 029615; RRID: IMSR_JAX:029615
Mouse: $Asxl1^{Y588X}$Tg	Our lab	
Mouse: NOD.Cg-$Prkdc^{scid}$ $Il2rg^{tm1Wjl}$/SzJ	The Jackson Laboratory	JAX: 005557; RRID: IMSR_JAX:005557
Recombinant DNA		
lentiCRISPRv2GFP	Addgene	Cat# 82416; RRID: Addgene_82416
lentiCRISPRv2GFP-sgKDM6B	This Paper	N/A
pLV-EGFP-U6-Scramble-shRNA	This Paper	N/A
pLV-EGFP-U6-GATA2-shRNA#1	This Paper	N/A
pLV-EGFP-U6-GATA2-shRNA#2	This Paper	N/A
pLV-EGFP-U6-MEIS1-shRNA#1	This Paper	N/A
pLV-EGFP-U6-MEIS1-shRNA#2	This Paper	N/A
